# Century-long timelines of herbarium genomes predict plant stomatal response to climate change

**DOI:** 10.1038/s41559-024-02481-x

**Published:** 2024-08-08

**Authors:** Patricia L. M. Lang, Joel M. Erberich, Lua Lopez, Clemens L. Weiß, Gabriel Amador, Hannah F. Fung, Sergio M. Latorre, Jesse R. Lasky, Hernán A. Burbano, Moisés Expósito-Alonso, Dominique C. Bergmann

**Affiliations:** 1 Department of Biology, Stanford University, Stanford, CA USA; 2 Howard Hughes Medical Institute, Stanford University, Stanford, CA USA; 3 Department of Biological Sciences, California State University San Bernardino, San Bernardino, CA USA; 4 Department of Biology, Pennsylvania State University, University Park, PA USA; 5 Department of Genetics, Stanford University, Stanford, CA USA; 6 Department of Developmental Biology, Stanford University School of Medicine, Stanford, CA USA; 7 Centre for Life’s Origins and Evolution, Department of Genetics, Evolution and Environment, University College London, London, UK; 8 Research Group for Ancient Genomics and Evolution, Department of Molecular Biology, Max Planck Institute for Biology, Tübingen, Germany; 9 Department of Plant Biology, Carnegie Institution for Science, Stanford, CA USA; 10 Department of Global Ecology, Carnegie Institution for Science, Stanford, CA USA; 11 Present Address: Department of Plant and Microbial Biology, University of California, Berkeley, CA USA; 12 Present Address: Department of Integrative Biology, University of California, Berkeley, CA USA; 13 Present Address: Howard Hughes Medical Institute, University of California, Berkeley, CA USA

**Keywords:** Plant evolution, Natural variation in plants, Climate-change impacts, Evolutionary developmental biology

## Abstract

Dissecting plant responses to the environment is key to understanding whether and how plants adapt to anthropogenic climate change. Stomata, plants’ pores for gas exchange, are expected to decrease in density following increased CO_2_ concentrations, a trend already observed in multiple plant species. However, it is unclear whether such responses are based on genetic changes and evolutionary adaptation. Here we make use of extensive knowledge of 43 genes in the stomatal development pathway and newly generated genome information of 191 *Arabidopsis thaliana* historical herbarium specimens collected over 193 years to directly link genetic variation with climate change. While we find that the essential transcription factors SPCH, MUTE and FAMA, central to stomatal development, are under strong evolutionary constraints, several regulators of stomatal development show signs of local adaptation in contemporary samples from different geographic regions. We then develop a functional score based on known effects of gene knock-out on stomatal development that recovers a classic pattern of stomatal density decrease over the past centuries, suggesting a genetic component contributing to this change. This approach combining historical genomics with functional experimental knowledge could allow further investigations of how different, even in historical samples unmeasurable, cellular plant phenotypes may have already responded to climate change through adaptive evolution.

## Abstract (web summary)

Exploring genomic data from contemporary and 191 *Arabidopsis thaliana* herbarium specimens collected over 193 years, the authors identify signs of local adaptation in regulators of stomatal development in contemporary samples from different geographic regions, then use functional scoring to identify a genetic component contributing to this change.

## Main

Ongoing drastic increases in atmospheric CO_2_ concentrations (subsequently [CO_2_]) and the resulting changes in global temperatures and drought severity are altering our environment^1^. Dissecting whether and how plants respond to this will be key to understanding plants’ potential for adaptation to climate change, and to developing strategies to increase their chances of survival. Several phenotypic trends observed in large numbers of plant species and across continents are being reported, including the acceleration of flowering^2,3^ and other life history events^4^, the increase in photosynthesis^5^ and the decrease in the number of stomatal pores in plant leaves^6^. However, it has thus far been difficult to resolve whether these trends reflect plastic phenotypic changes or result from evolutionary genetic change^7^. Stomata are one of the plant structures that are most directly relevant to multifactorial climatic changes, as these surface pores, essential for survival and productivity, are major contributors to plants’ water-use efficiency (WUE): the ratio between CO_2_ uptake for photosynthesis (*A*) and the release of O_2_ and loss of water vapour with transpiration (*E*; reviewed in ref. ^8^). Optimizing WUE to environmental conditions can improve plant fitness and yield when plant growth and cooling through transpiration are balanced with minimal water loss. WUE is partially fine-tuned through variation in stomatal size and density (that is, the amount of stomata per plant surface area^9–11^). This stomatal variation can represent temporary plastic responses or result from evolutionary genetic change over generations that produces local adaptation^12–14^.

A powerful way to differentiate between plastic and evolutionary plant responses to climate is to study different populations of a single species collected across geographic climatic gradients in combination with genetic analyses and common gardens^15^. Both stomatal size and density of natural populations of *Arabidopsis thaliana* measured in controlled environments correlate with climate variables of the populations’ geographic origin, indicating a potential genetic basis for their climate responses^16^. In *Arabidopsis*, lower stomatal density typically follows increasing [CO_2_] and temperature, while higher stomatal densities and drought-adjusted higher WUE result from decreased humidity. Stomatal size is generally anti-correlated with stomatal density^16–20^. Given this connection of stomatal variation with climate gradients within a species, we would expect the past ~200 years of anthropogenic global change to have impacted such plant stomatal variation in complex unknown ways. Collections of pressed and dried specimens of different species are witnesses of plant responses to the [CO_2_] increase from ~280 parts per million (p.p.m.) in 1750 to 419 p.p.m. in 2022 (August measurement; see trends at www.climate.gov and climate.nasa.gov), or the current global maximum temperature anomaly of approximately +1 °C (climate.nasa.gov), and, with that, we can track adaptation to climate change as it happens (reviewed in ref. ^21^).

Plant responses themselves can help infer historical climate trends. Measurements of stomatal densities in fossils indicate [CO_2_] changes over geological time^22–25^, and over the recent anthropogenic climate change, decreases in stomatal densities preserved in herbaria already reflect the industrialization-related increases in [CO_2_] (ref. ^6^). Such analyses have thus far never extended beyond phenotypic quantification to also assess genetic changes underlying these potentially adaptive responses, mainly because fossil records lack quantifiable DNA. Now, sequencing of herbarium specimens can address this gap by directly exploring joint timelines of phenotypic and genotypic responses to climate change (for example, refs. ^26–30^).

Stomata are a unique system to use such timelines to understand plants’ adaptive potential and its mechanisms, as their genetic pathway is dissected in minute detail in *A. thaliana* (for example, reviewed in refs. ^8,31,32^): From the sequential interactions of indispensable transcription factors that regulate cell production, fate and patterning to external regulators that fine-tune stomatal development in response to environmental and physiological stimuli. This knowledge provides a crucial advantage when investigating a genetic basis of potential stomata involvement in climate adaptation. It allows selective study of genetic variants in already-validated causal genes and can complement genome-wide association ‘discovery’ approaches, which so far have yielded highly polygenic signals elsewhere in the genome that explain part of the observed phenotypic variation but are not easily connected to specific effects on phenotypes or functions^16,33^. We can start accounting for this polygenic complexity by integrating genetic information and a functional understanding of the entire genetic developmental pathway and directly ask whether and how known stomatal development genes may promote adaptation.

In this Article, we use historical specimens as ‘witnesses’ of the ongoing climate change together with molecular genetics knowledge to ask: Can herbaria reveal climate change adaptation in the genetic pathways of essential plant features such as stomata? Can combination of historical and modern genomes with genes’ known phenotypic effects circumvent currently lacking historical phenotyping to predict plant change?

## Results and discussion

### Stomata genes show a purifying natural selection signal

To investigate how stomata, or stomatal development, has responded to climatic change, we created a new temporal dataset^34,35^ of 191 broadly geographically distributed historical samples, covering the time period from 1817 to 2010 (Supplementary Fig. 1a and Supplementary Table 1), and paired it with the contemporary *A. thaliana* 1,001 genomes resource^36^. So-called ancient DNA, retrieved from the historical herbarium specimens, was authenticated following the field’s standards (for example, ref. ^28^). Samples show the expected patterns of age-related DNA fragmentation (merged fragments’ median size of 98 bp; Supplementary Fig. 1b,c) and damage, that is, cytosine deamination (reflected as C-to-T substitutions in sequencing data; 0.7–4%; Supplementary Fig. 1d), accumulated particularly at DNA molecules’ termini. As previously shown, deamination of a fragment’s first base is highly correlated with the year of sample collection (one-sided Pearson’s correlation test, correlation coefficient *r* = -0.589, *P* = 1.693 × 10^−15^; Supplementary Fig. 1e), a pattern that reflects post-collection ageing of the specimens as a primary cause of DNA damage but whose strength depends on multiple additional factors such as plant specimen collection and storage conditions^37,38^.

**Fig. 1:**
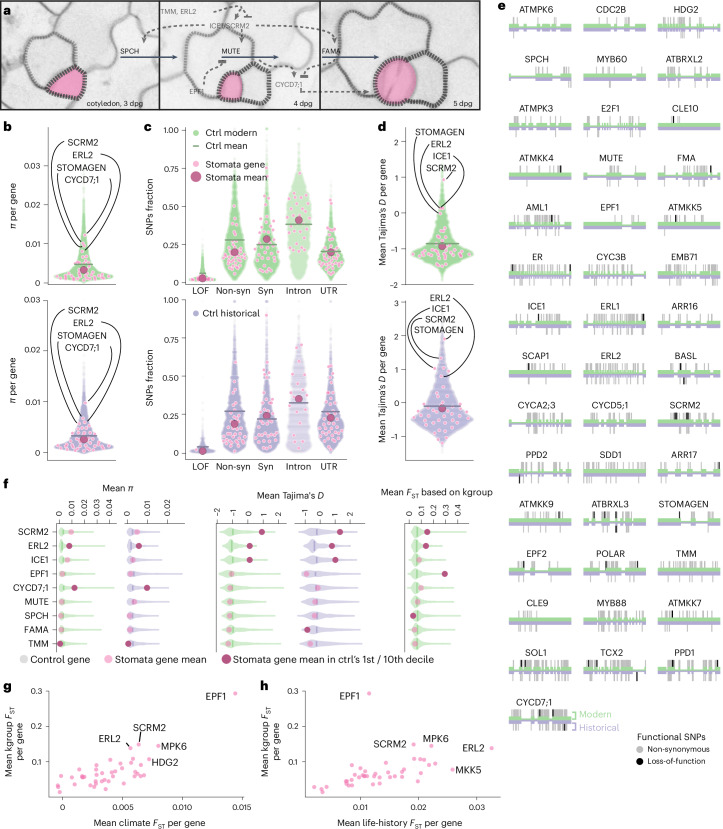
Conserved core stomata genes and regulatory genes show local adaptation signals. **a**, Stomatal development in *A. thaliana* (simplified, example regulators in grey, central (core) transcription factors in black. Stomata false-coloured in magenta. Cotyledons imaged at 3, 4 and 5 days post germination. **b**, Genetic diversity in stomatal genes is significantly lower than in length-matched control genes (gene names mark outliers; nucleotide diversity *π* per gene, empirical *P*_mod_ = 0.004, *P*_hist_ = 0.046). **c**, Significantly fewer SNPS in stomatal genes are putative LOF or non-synonymous than in the control genes (empirical *P*_mod_^non-syn^ = 0, *P*_hist_^non-syn^ = 0.002, *P*_mod_^LOF^ = 0, *P*_hist_^LOF^ = 0.047; Supplementary Table 3). Ctrl, control; Non-syn, non-synonymous. **d**, Mean per-gene Tajima’s *D* indicates selection signals. Stomatal gene group is not significantly different (*P*_mod_, *P*_hist_ > 0.1) from the control, but several genes are outliers (labelled). Significance tests for panels **b**–**d** asked whether means of the group of stomatal genes were outliers compared with the means of 1,000 control gene groups. Purple for historical, green for modern datasets, horizontal line indicates full control dataset’s mean. Magenta circles for stomatal genes, large dark circles for stomatal gene group mean. **e**, LOF (black) and non-synonymous (grey) SNPs in the 43 focus genes in historical (bottom, purple) and modern (top, green) dataset. **f**, Mean per-gene values for nucleotide diversity *π*, Tajima’s *D* and *F*_ST_^kgroup^ values for outlier genes, compared with conserved stomatal factors SPCH, MUTE and FAMA (for all 43 genes, see Supplementary Figs. 2 and 3 and Supplementary Table 2). *F*_ST_^kgroup^ is calculated for populations defined by whole-genome genetic variation (from ref. ^49^). Gene values are displayed as transparent pink circles on violin plots representing distribution of values for the respective length-matched control genes, vertical line indicating distribution’s 0.5 quantile. Solid pink circles indicate that the gene mean value lies within the 1st/10th decile of the control distribution. **g**,**h**, Stomatal gene differentiation as mean *F*_ST_ per gene, with *F*_ST_^kgroup^ (*y*-axis), compared with *F*_ST_ for populations clustered by climate of origin (precipitation, temperature, BIO4 and BIO15, Bioclim dataset; ref. ^50^) (**g**) and life-history traits (data; ref. ^51^) (**h**). Genes with the highest *F*_ST_ values across the three analyses are labelled.

Because stomatal density differences have been observed across geographic regions of *A. thaliana* and these changes are partially heritable^16,33^ we aimed to depict genetic variation in known genes of the stomatal pathway, which could constitutively alter stomatal densities. We surveyed the literature and defined a set of 43 genes experimentally validated to be involved in stomata development (Supplementary Table 2) that focuses on genes mediating the cell divisions and fate transitions central to stomatal development (Fig. 1a). Aiming to specifically inquire whether the central developmental pathway itself creates constitutive stomatal density differences that can undergo positive selection, we excluded genes that are predominantly characterized as environmental sensors affecting stomatal stress responses.

We hypothesized that if there was an increased genetic diversity in these 43 stomatal genes, it might reflect broad variation in stomatal size and density (the number of stomata per plant surface area) that may have allowed local adaptation to the different environments encountered by the species across its geographic range. To investigate this, we examined our modern and historical genomes as ‘snapshots’ of the species’ current and past ~200 years of global genetic diversity (Fig. 1b,c). Overall, expectedly, we found fewer single-nucleotide polymorphisms (SNPs) in the 191 historical than the 1,135 modern samples. This is consistent with the historical dataset’s much smaller size and stringent SNP-calling that has to account for characteristics and sequencing challenges intrinsic to historical DNA (Supplementary Fig. 1b–d; for example, ref. ^28^). In addition, a modern increase in SNP numbers is also consistent with a recent human-linked population expansion of the species^39,40^. To compare genetic diversity in these genes in the context of the broader genome, we generated 1,000 sets of 43 randomly drawn control genes, with a gene length distribution matched to that of the 43 stomatal genes. In comparison to this control, stomatal genes harbour drastically reduced genetic diversity, as estimated by Watterson’s *θ* (Supplementary Fig. 2; ref. ^41^; *P*_mod_ = 0.006, *P*_hist_ = 0.069) and lower nucleotide diversity *π* (Fig. 1b and Supplementary Fig. 2; ref. ^42^, *P*_mod_ = 0.004, *P*_hist_ = 0.046). This would be expected if purifying selection is purging genetic variation in the developmentally important stomatal genes.

Analyses of non-synonymous and synonymous SNPs in stomata and control genes further show that in stomata genes there are significantly fewer variants annotated as potentially affecting protein function, which we expect to be generally detrimental (partial or full loss of function (LOF) from missense, frameshift or gain of stop codon), compared with likely neutral variants (located in introns, untranslated regions (UTRs) or degenerate codons; annotation with SnpEff^43^; *P*_mod_^non-syn^ = 0.011, *P*_hist_^non-syn^ = 0.003, *P*_mod_^LOF^ = 0.003, *P*_hist_^LOF^ = 0.049; Fig. 1c and Supplementary Table 3). Despite the overall low variation, in the modern data all but one stomatal genes do harbour non-synonymous variation (from 4 (*CLE10*, AT1G69320) to 124 (*CYCD7;1*, AT5G02110)), and some even contain LOF variants at low frequency (from 1 to 4, in 23 out of 43 genes; Fig. 1e and Supplementary Table 2). We hereafter refer to non-synonymous and LOF variants jointly as putatively functional variation. As expected, the transcription factors that are essential for stomatal development are among the genes with the lowest genetic variation (*SPCH* = 0.006 SNPs per bp, *MUTE* = 0.008 SNPs per bp, *FAMA* = 0.008 SNPs per bp; Fig. 1e, genes ordered by modern data-based SNPs per bp, from left to right, top to bottom). We concluded that stomata genes generally seem to be under purifying selection—especially master transcription factor regulators that lack variation and are unlikely involved in local adaptation—although some genes still harbour non-synonymous variants at low frequency that could have strong phenotypic effects.

This led us to think of the stomatal pathway as composed of two main groups of genes. One group represents highly conserved essential core genes, where the functional loss of any single gene drastically affects stomatal morphology and density or is even lethal^44–46^. Here, examples are the above-mentioned non-redundant master transcription factors SPEECHLESS (AT5G53210), MUTE (AT5G53210) and FAMA (AT3G24140). The second group consists of their direct and indirect regulators that fine-tune the core pathway’s activity and outcome, including duplicated, redundant genes in the pathway that as pairs—but not individually—are similarly essential (Fig. 1a). As loss or functional changes of single ‘regulator’ genes have on average less impact on overall plant development, they are under less evolutionary constraint and thus may be more likely to harbour genetic variation available for positive selection.

### Local adaptation signals in stomata regulator genes

If any of the functional natural variation in stomatal genes is adaptive, population genetics statistics might detect potential signals of selection and population differentiation. We used a battery of Tajima’s *D* and *F*_ST_ statistics among populations grouped by population history, phenotypes or climates. Tajima’s *D*, which aims to distinguish between neutrally evolving polymorphic sites and those that may be under positive or balancing selection^47^, does not significantly differ between the groups of stomatal and control genes (Fig. 1d; *P*_mod_, *P*_hist_ > 0.1). However, when comparing each individual stomatal gene with its specific control distribution, we saw a negative Tajima’s *D* value for the genes encoding essential core transcription factors SPCH, MUTE and FAMA (Fig. 1f, Supplementary Fig. 2 and Supplementary Table 2). This fits with their key roles as necessary and sufficient drivers of stomatal development^44–46,48^. By contrast, we identified several outliers among the regulatory genes with both high *π*, high positive Tajima’s *D* and high population differentiation measured by Wright’s *F*_ST_ between geographically separated *A. thaliana* populations (*F*_ST_^kgroup^, based on *k* = 11 groups^49^). The combination of high species-wide Tajima’s *D* and high cross-population *F*_ST_ may reflect above-average differentiation of alleles between populations relative to within-population differentiation, and maintenance of multiple alleles of the same gene that may be involved in local adaptation. We find indications of this in several genes, all among the 10% highest values of the control distribution for either one or both Tajima’s *D* and *F*_ST_^kgroup^ (Fig. 1f and Supplementary Fig. 2): *EPF1* (AT2G20875), *ERL2* (AT5G07180)—which also has the second highest *π* after *CYCD7;1*, *ICE1* (AT3G26744), *SCRM2* (AT1G12860) and *STOMAGEN*/*EPFL9* (AT4G12970).

If these outlier genes are indeed involved in local adaptation, the distribution of their genetic variation might follow environmental (climate) gradients such as variability in temperature and humidity^16,19^. We tested this by grouping samples into populations either based on their collection locations’ precipitation and temperature seasonality (BIOCLIM 4 and BIOCLIM 15; ref. ^50^) or based on plant life history traits typically aligned with climate, such as the timing of germination or flowering for optimal survival and reproductive success in a given environment (Fig. 1g,h and Supplementary Fig. 3; ref. ^51^). Using *F*_ST_ statistics, we then assessed genetic differentiation between the resulting populations. Despite variation in the absolute *F*_ST_ values, there is clear overlap in the genes that are most differentiated between populations, independent of using population ancestry groups to compute a traditional *F*_ST_, or climate or life history to delineate said populations, with *SCRM2* and *EPF1* among the top four climate differentiators (Fig. 1g and Supplementary Fig. 3) and *SCRM2* and *ERL2* in the top four life-history genes (Fig. 1h and Supplementary Fig. 3; see also Supplementary Text 1 and Supplementary Fig. 6b–d for stomatal gene differentiation over time). A single LOF of the top four adaptation candidates *SCRM2*, *ERL2*, *EPF1* and *ICE1* tends to minimally affect plant development beyond the stomatal context^52–54^, with the exception of *ICE1*’s role in endosperm breakdown and embryo development^55^. Interestingly, the basic Helix-Loop-Helix transcription factors and paralogs *ICE1* (*SCRM*) and *SCRM2*, both involved in cold tolerance, act redundantly as direct interaction partners and expression regulators of *SPCH*, *MUTE* and *FAMA*^54^. This fits the expectation of strong purifying selection acting in indispensable master transcription factors and local adaptation through contribution of flexible regulators.

### Functional score recovers phenotype changes across geography

**Fig. 2:**
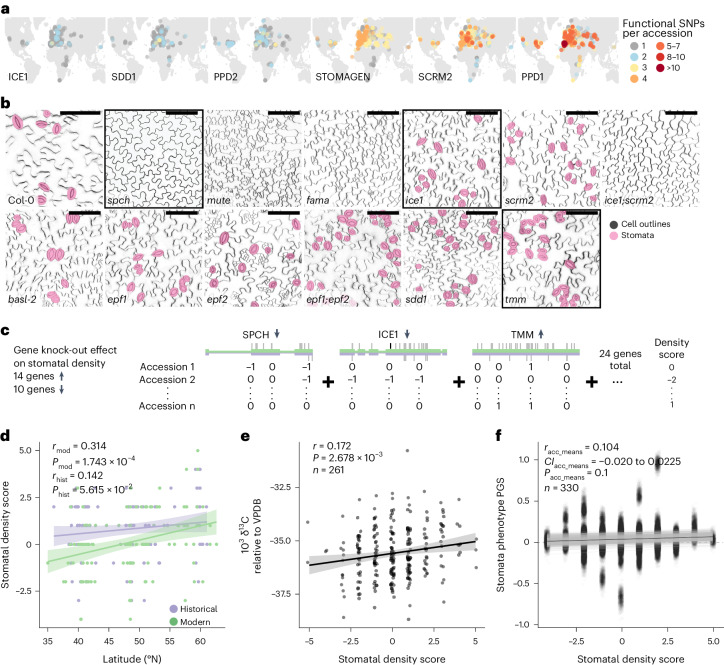
Functional score predicts stomatal density patterns. **a**, Geographic distribution of functional (non-synonymous, LOF) SNP accumulation in historical and modern samples for six genes with the overall highest amounts of putatively functional SNPs, overlaid on a continental map. Colour gradient from grey to dark red indicates samples with 1 to over 10 functional SNPs. **b**, Gradient of stomatal density differences resulting from loss of major stomatal development genes visualized with confocal microscopy. Stomata are false-coloured in magenta; scale bars, 100 µm. Black frames around three example genes used in **c**. **c**, Schematic overview detailing the generation of the experimentally informed stomatal density score. Of 24 genes with known effect on stomatal density, loss of 14 increases and loss of 10 decreases stomatal density. Putatively functional SNPs are assigned a ‘−1’ when located in genes whose loss decreases density and a ‘+1’ in genes whose loss increases density. Density scores for each historical and modern sample are calculated as the sum of these values across the 24 genes, counting a single functional SNP per gene. **d**, Linear regression (±s.d.) of the stomatal density score with paired samples’ latitude of origin, separated into historical (purple) and modern (green) samples (for each *n* = 126, one-sided Pearson’s correlation test *P*_mod_ = 1.743 × 10^−4^, *P*_hist_ = 5.615 × 10^−2^, correlation coefficient *r*_mod_ = 0.314, *r*_hist_ = 0.142). Analyses exclude samples from North America and the African continent. **e**, Correlation (±s.d.) of the density score with the δ^13^C measurement, a proxy of WUE, in 261 *A. thaliana* accessions. This is based on isotope amount ratios of stable carbon isotopes ^13^C/^12^C, expressed as ‰ against the Vienna Pee Dee Belemnite (VPDB; ref. ^111^) standard (one-sided Pearson’s correlation test *P* = 2.678 × 10^−3^, correlation coefficient *r* = 0.172; δ^13^C (stable carbon isotope ratio) data from ref. ^16^). **f**, Correlation (±s.d.) of the stomatal density score with genome-wide association-based traditional PGS for stomatal density (one-sided Pearson’s correlation test, positive correlation 997/1,000 re-trainings, 141/1,000 significant one-sided Pearson’s correlation tests with *P* < 0.05, correlation coefficient *r*_median_ = 0.092; stomatal density data from ref. ^16^).

Although the stomatal development pathway at first glance may appear relatively simple, leaf stomatal density is a complex trait (Supplementary Text 2). It is likely affected by a combination of (environmental) factors and complex fine-tuning of the aforementioned core and regulatory genes and likely many others. Genome-wide associations of stomatal density thus far yielded moderate heritabilities across many genomic regions, explaining only small fractions of the variation with high uncertainty on specific causal variants^16^. As numerous previous studies provide causal evidence that well-known stomatal genes affect the trait, we study these genes’ SNPs located in coding regions where we infer protein amino-acid composition change, that is, non-synonymous changes leading to loss or gain of function. If such putatively functional SNPs are then under climate-driven natural selection, we should expect them to follow geographic and historical climate gradients and that the non-reference functional variants in multiple genes appear in concerted fashion^56^. Indeed, when visualizing the distribution of such variants in six genes with the most putatively functional SNPs, they appear geographically segregated (Fig. 2a).

In our set of 43 stomatal development genes, 24 have confirmed phenotypes in increasing or decreasing abaxial leaf stomatal density (mostly shown by knock-out mutant analysis; see exemplary phenotypes in Fig. 2b; refs. ^44–46,48,54,57–69^). We combined this information on functional variation in the 24 stomatal density genes in our historical and modern samples with the genes’ putative effects on increasing or decreasing stomatal density. With this genetic and phenotypic information, we devised a simple cumulative ‘stomatal density score’ to indicate increasing (positive) or decreasing (negative score) stomatal density compared with the baseline accession Col-0 (in which functional variation is typically characterized). This functional score is conceptually similar to polygenic scores but is based on summing over putatively functional variation of genes with known increase/decrease effects on a trait (for distinction from genome-wide association (GWA), see also Supplementary Text 3). Putatively functional SNPs are assigned a ‘−1’ in genes whose knock-out decreases stomatal density (10 genes) and a ‘+1’ in genes whose knock-out increases stomatal density (14 genes; Supplementary Table 4), where genes with multiple SNPs are only counted once (Fig. 2c). This recovered a gradient of ‘stomatal density scores’ (Fig. 2d–f).

We found that this density score significantly correlates with modern samples’ latitude of origin, which in turn dictates life cycle length (Fig. 2d; one-sided Pearson’s correlation test *P*_mod_ = 1.743 × 10^−4^, *P*_hist_ = 5.615 × 10^−2^, correlation coefficient *r*_mod_ = 0.314, *r*_hist_ = 0.142; correlation consistently significant upon population structure correction; Supplementary Text 4). In fact, experimentally measured stomatal density also follows a latitudinal trend attributed to *A. thaliana*’s ecological and life-history adaptations to latitudinal climate gradients (Fig. 2d and Supplementary Fig. 4a,b; refs. ^16,51,70^). Despite the score being derived from simple functional single-gene knock-out experiments, we found that the score correlates positively with a measure of integrated life-time WUE based on CO_2_ exchange and water loss (Fig. 2e; δ^13^C, difference in the ratio of ^13^C to ^12^C compared to a standard; one-sided Pearson’s correlation test *P* = 2.678 × 10^−3^, correlation coefficient *r* = 0.172). A positive trend was found with stomatal density measured in modern *A. thaliana* populations (Supplementary Fig. 4c; δ^13^C and stomatal density data from ref. ^16^). Permuting the functional (positive or negative) effects of the 24 genes and recomputing the density score removed all significant relationships above (Supplementary Fig. 4d,e), indicating that the density score is not a result of internal dataset biases or population structure. Finally, we compared our functionally derived density score with a traditional GWA-based polygenic score (PGS; refs. ^71,72^) based on published stomatal density data^16^. PGS trained on 80% of the data explain on average 9.2% of the variance in the reserved 20% of phenotype data (positive correlation in 1,000 out of 1,000 re-trainings, 745 out of 1,000 significant one-sided Pearson’s correlation tests with *P* < 0.05, correlation coefficient *r*_min–max_ = 0.010-0.561, *r*_median_ = 0.304; Supplementary Fig. 5a,b). They also correlated with the functionally derived density score (on average 0.9% of the density score’s variance explained; positive correlation in 997 out of 1,000 re-trainings (genome-wide), 141 out of 1,000 significant one-sided Pearson’s correlation tests, *P* < 0.05, correlation coefficient *r*_min–max_ = −0.020–0.204, *r*_median_ = 0.092; Fig. 2f and Supplementary Fig. 5c (only stomata genes); correlations with experimentally measured density and density score lost upon randomization of the phenotype–genotype associations in the training dataset; Supplementary Fig. 5d,e and Supplementary Table 5).

### Functional score predicts reduction of stomatal density

**Fig. 3:**
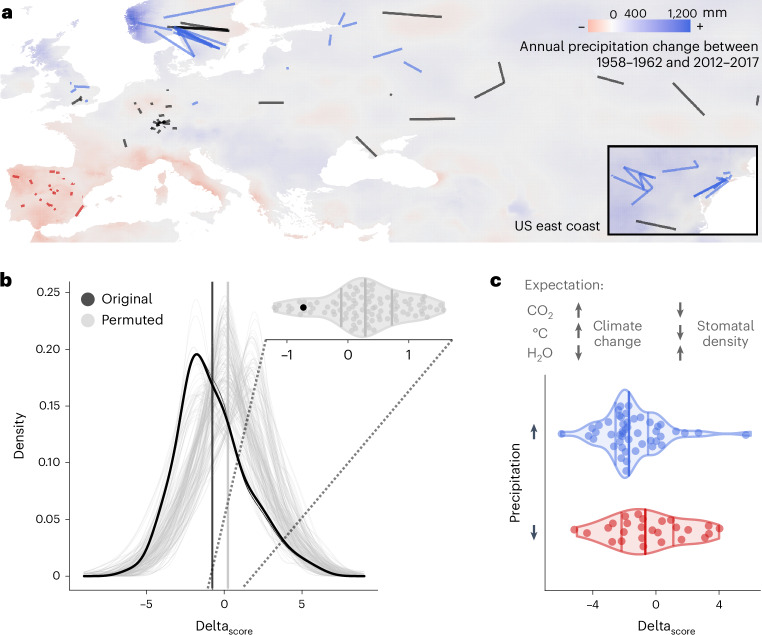
Stomatal density decrease over time fits climate change expectations. **a**, Map with historical and modern sample pairs as used for stomatal density change analyses (in **b** and **c**). Connecting lines between sample pairs are coloured by the precipitation change in the sample locations; red indicates a significant decrease in precipitation from 1958 to 2017 in both the historical and modern sample location, blue indicates a significant increase in precipitation, and black no significant change or different changes in paired locations. Background colour gradient indicates change in the mean annual precipitation between 1958–1962 and 2012–2017, with colours as above. Inlay shows sample pairs located on the North American East Coast. **b**, Distribution of per sample-pair calculated difference in stomatal density scores (delta_score_ = modern − historical) for original data (black) and 100 permutations (grey) between genes and their assigned effect (decrease/increase) on stomatal density, with genetic variation itself remaining un-permuted; mean delta_score_ = −0.730, Wilcoxon signed rank test, *P* < 2.2 × 10^−16^. Density distribution means are marked by solid black and grey vertical lines. Violin plot of the distribution means, with the non-permuted mean delta_score_ lower than 92/100 permutations. **c**, Expected effects of climate-change-related shifts in [CO_2_], temperature and water availability on stomatal density (based on published experiments, for example, refs. ^17–20^). Change in the stomatal density score (delta_score_ = modern − historical) in sample pairs with significantly increased (blue, *n* = 48) or decreased (red, *n* = 26) precipitation in geographic locations of origin (excluding sample pairs where precipitation did not change significantly or where a pair’s locations did not change in the same way). Horizontal lines indicate 0.25, 0.5 and 0.75 distribution quantiles. Increased, but not decreased, precipitation is significantly associated with decreased delta_score_ (linear regression, delta_score_ ≈ precipitation_directionality_, *P*_incr_precipitation_ = 0.024, *P*_decr_precipitation_ = 0.889, *P*_model_ = 0.045; Supplementary Table 9). Analyses include samples from North America and exclude samples from the African continent as well as pairs between island and mainland samples.

Ultimately, we aim to understand how stomatal variation in *A. thaliana* may have changed over the past centuries of climate change. For this, we calculated our stomatal density score on historical genomes. To avoid geographical biases past and present, we made comparisons within a subset of historical and modern samples, paired based on geographic proximity (126 pairs, minimum–maximum distance = 0.5–495 km, mean = 139 km; median = 61 km; Pearson’s correlation of sample pairs’ latitudes of origin, *r* = 0.990, *P* < 2.2 × 10^−16^; Fig. 3a, Supplementary Fig. 6a and Supplementary Table 6). The historical set for these comparisons ranged between 1817 and 2002 (mean collection year 1923) and the modern set between 1992 and 2012 (mean 2001), with a mean age difference of 79 years in historical–modern sample pairs (from 1 to 182 years). Analyses included only shared SNPs across historical and modern data, to avoid biases related to dataset-specific ‘private’ SNPs.

Before calculating temporal trends, we established that the stomatal density score correlation with latitude remains consistent also in the historical dataset, suggesting patterns of local adaptation have long been established in the species (see historical trends in Supplementary Fig. 4a,b,d,e). We then calculated the difference in density score (delta_score_) for each individual historical–modern geographic sample pair (Fig. 3a). The resulting distribution of score changes corroborates previous results that suggest a decrease in stomatal density in modern samples (black distribution, mean delta_score_ = −0.730, Wilcoxon signed rank test, *P* < 2.2 × 10^−16^; Fig. 3b and Supplementary Fig. 7d). This decrease was robust to reanalyses with population group subsampling by removal of samples along a latitudinal gradient or of modern samples with uncertain collection dates (mean delta_score_^noUS^ = −0.433, delta_score_^noUS_noSpain^ = −0.423, delta_score_^noScandinavia^ = −0.745, delta_score_^noUncertainty^ = −0.717, Wilcoxon signed rank test, all *P* < 1.25 × 10^−15^; Supplementary Fig. 7a–c, Supplementary Table 7 and 8 and Supplementary Text 5). Recalculated temporal trends using permuted positive and negative stomatal density gene effects did not recover the originally predicted decrease in stomatal density. This suggests that the trend is not due to confounding structure of the data or population history but rather results from the coordinated gain or loss of non-synonymous nucleotide variation in positive and negative stomatal density regulator genes (Fig. 3b and Supplementary Fig. 7a–d, grey distributions).

Such a putative decrease in stomatal density over the past century would fit the expectation based on experimental evidence showing that increases in [CO_2_] and temperatures lead to leaves with lower stomatal density^6,17,20,23,25,73,74^. However, while [CO_2_] and temperature have mostly increased, climate change has also altered weather patterns and heterogeneously increased or decreased precipitation depending on the region on Earth^1^. Because experiments simulating decreased water availability show directly opposite stomatal responses (that is, density increases with less water availability; refs. ^18,75^), we wondered whether we could use the differential changes in precipitation as a ‘natural counterfactual’ experiment to validate our genetic prediction of stomatal changes over time. We thus conducted a sensitivity analysis, grouping sample pairs based on their temperature and precipitation trajectories and magnitudes of change over the past 60 years (Fig. 3a,c). Sample pairs from locations with increased precipitation are significantly more likely to have decreased stomatal density scores over time (Fig. 3c, Supplementary Table 9 and Supplementary Fig. 7e; almost 2.5-fold odds of stomatal decrease with Fisher’s exact test, odds_ppt_high_ = 2.291 (ppt = precipitation), *P*_ppt_high_ = 0.05, and consistent results for the same analysis with less stringent filters in Supplementary Fig. 7f; ref. ^76^; Supplementary Table 10 and Supplementary Text 5). Locations with decreased precipitation, a change counteracting the expected effects of increased [CO_2_] and temperature, showed a (non-significant) trend of stomatal density increase (Fisher’s exact test, odds_ppt_low_ = 0.589, *P*_ppt_low_ = 0.283, Supplementary Table 10; for example, refs. ^18,75^). This observation is consistent with the hypothesis that *A. thaliana* under drought would favour more and smaller stomata, as these open and close more rapidly.

Despite the spatial sample pairing and climate splits in our analyses, temporal trends in stomatal density score changes might still contain residual biases such as population structure. We therefore conducted a series of analyses including fitting the first three main genomic principal components (PCs) to capture population structure and describe the consistent stability of various estimates (Supplementary Text 5). The signal of lowering average stomatal density remains after population structure correction (*P*_model2_ = 0.001; Supplementary Text 5 and Supplementary Table 9), and also the more pronounced decrease in stomatal density in regions with increased precipitation is mostly consistent after corrections for each genomic PC axis (*P* = 0.034, 0.081, 0.019, after PC1, PC2, PC3 corrections, while no variable is significant with a full model; Supplementary Text 5 and Supplementary Table 9). Taken together, despite the noise inherent to naturally evolving structured populations and the counteracting effects of [CO_2_] and temperature versus precipitation, there is a consistent signal-to-noise ratio in the identified trends of decreasing stomatal density. These trends correspond well with both experimental and historical observations of stomatal density responses to the climate variation connected with global change.

## Conclusions and outlook

Global change has led to rapid and drastic changes of multiple climate parameters—atmospheric CO_2_ concentration, temperature, precipitation—that have a strong impact on plant development. Here we studied responses of *A. thaliana* leaf stomatal development to anthropogenic climate change, using historical herbarium genomes as witnesses of this multi-factorial ‘global change experiment’ (for example, refs. ^6,77^). Despite the overall high conservation of stomatal development genes, our integrative approach allowed us to identify evolutionary signals in several of these genes that are consistent with local adaptation across *A. thaliana*’s geographic range, suggesting that the well-described stomatal development pathway itself could also evolve to climate change conditions. We developed a novel functional score based on functional and experimental molecular knowledge of stomatal development genes that agrees with a historical trend of stomatal density decrease in *A. thaliana*, classically observed across species but of unknown genetic basis^6,24^. This trend is consistent with experiment-based expectations and climate counterfactuals but may certainly be influenced by additional factors beyond climate change itself. While evolutionary processes can be fast (for example, refs. ^40,78,79^), our analyses show that even with hundreds of historical genomes, the described trends of putative adaptive evolution are significant yet close to the detection limit above the noise of genetic drift, phenotypic plasticity, counteracting climatic factors and methodological idiosyncrasies, as seen with the re-analyses of different geographical subsets of the data. Our discovery will stimulate follow-up investigations such as studying the molecular mechanisms of how these genes promote adaptation—now enabled, for instance, by single base-editing with CRISPR (clustered regularly interspaced short palindromic repeats) to recreate historical variants^80,81^—characterizing cellular phenotypes directly from historical specimen tissues using customized microscopy techniques (for example, ref. ^82^) and generating denser timelines of historical genomes possible with high-throughput ancient DNA technologies^29,83^. The functional historical genomics approach presented here adds an exciting avenue to leverage the power of genomics to reconstruct phenotypic impacts of climate change on species, even for those phenotypes that are cellular or sub-cellular. Ultimately, our approach could help uncover complex responses involving WUE, photosynthetic capacity or drought resistance that are not preserved or measurable in historical collections. Disentangling this will be key to understanding plants’ past and future adaptive potential and design targets for engineering plants for the future.

## Methods

### Sequence data

#### Contemporary data

Published^43^ *A. thaliana* SNPs^36^ were annotated (TAIR10_GFF3_genes_transposons.gff; ref. ^84^). We extracted ‘genic’ SNPs using bash, BCFtools v1.10.2^85^, PLINK v1.90b6.16 64-bit^71,86^ and R^87^ within RStudio v1.2.1335 (ref. ^88^). Samples lacking geographic origin coordinates or collection year were excluded as indicated.

#### Herbarium data

##### Sample processing and sequencing

African (*n* = 9), North American (*n* = 33), German (*n* = 34) and broadly geographically distributed (*n* = 204) *A. thaliana* historical whole-genome sequences were downloaded^34,35,40,89^.

Historical sequencing was processed as described^28,90^. We mapped the merged reads(Adapterremoval v2.3.1; ref. ^91^; BWA v0.7.15-r1140; ref. ^92^) to TAIR10 (ref. ^84^), filtered for mapping quality ≥20 (Samtools v1.9; ref. ^85^), removed PCR duplicates (DeDup v0.12.8; ref. ^93^) and confirmed the authenticity of historical samples with fragmentation (Samtools v1.9; ref. ^85^; median merged fragment length of 98 bp, insert sizes for sheared, unmerged fragments of 234 bp) and deamination patterns (MapDamage v2.2.1; ref. ^94^; Supplementary Fig. 1). For double-processed (sheared and unsheared) broadly geographically distributed samples^35^, we concatenated the merged fraction of unsheared samples with the merged and unmerged fractions of sheared samples.

Based on sequencing and mapping statistics from Samtools (stats), DeDup and MapDamage, we defined quality thresholds. We retained samples with >1,000,000 bp or > 10,000 reads sequenced, >95% of reads mapped, a duplication rate <0.3 and an error rate <0.02 for de novo SNPcalling and excluded samples with >0.5 missing genotypes, resulting in a total of 191 samples with an ~1.5–39× genome coverage (mean 7.1×, median 5.9×). We did not differentiate between ‘regular’^34,35^ and UDG-treated libraries^40,89^. Samples lacking geographic origin or collection year were excluded as indicated.

##### De novo SNPcall

For genome-wide de novo calling of SNPs in the historical dataset, we created and indexed a reference dictionary with TAIR10^84^ using Picard’s CreateSequenceDictionary (Picard v2.18.29-0; ‘Picard Toolkit.’ 2019. Broad Institute, GitHub Repository. https://broadinstitute.github.io/picard/; Broad Institute) and samtools faidx (Samtools v1.9,^85^). We called variants across our herbarium samples by calling haplotypes for individual samples (GATK HaplotypeCaller), combining .gvcf files into a single input file (GenomicDBImport) and calling variants de novo (not based on an existing SNPs set, GenotypeGVCF) following GATK best practice recommendations (gatk4-4.2.0.0.-0; ref. ^95^). Keeping only SNPs (vcftools --remove-indels, VCFtools/0.1.16; ref. ^96^), we either included only or excluded all sites present in the published 1001G vcf file (vcftools --positions^36^). To determine parameters for quality-based SNP filtering, we compared quality parameters in the two subsets and the full dataset (vcftools --gzvcf <infile.vcf.gz > --get-INFO QD --get-INFO FS --get-INFO ReadPosRankSum --get-INFO MQRankSum --get-INFO BaseQRankSum–out <outfile>) using R. Assuming that the distributions of quality parameter values of the 1,001-only dataset are representative for high-quality SNPs, we defined cut-offs for SNP filtering with vcflib (vcffilter -f DP > 22 & FS < .2 & ReadPosRankSum>(0–2) & ReadPosRankSum<2; vcflib/20161123-git,^97^). Briefly, DP refers to the combined depth of a site across all samples; FS indicates strand bias as estimated by Fisher’s exact test; and ReadPosRankSum compares the positioning of reference versus alternative alleles within a read, allowing to filter alleles biased to read ends. Subsequently, we excluded samples with missing call frequencies >50% and filtered for biallelic positions with PLINK (plink --mind .5; PLINK v1.90b6.16 64-bit^71,86^). We used PLINK to remove variants where <3 individuals carried the alternative allele (--mac 3) and samples with site missingness >15% (--geno .15; Supplementary Fig. 8). We used variant-based PCAs to visualize filters’ effects on samples’ genetic diversity and identify outliers (PLINK --pca <sample_number>). The resulting vcf files were annotated using SnpEff v5.0e^43^ and the TAIR10 *A. thaliana* reference genome^84^ and further annotated and subset as described above for contemporary data.

#### Joined historical and modern data

Before merging, the historical and modern datasets were individually filtered with PLINK for alleles with a minimum minor allele count of 3 and for samples with <15% site missingness (‘De novo SNPcall’). Datasets were then intersected using BCFtools (bcftools isec -n = 2, v1.10.2^85^) and filtered for shared SNPs (PLINK --extract <shared_SNPs>) to avoid dataset-specific biases. Gene-specific and control subsets were generated as described above.

For direct comparisons of historical and modern samples, we subset the datasets to 126 geographically matched sample pairs (‘Geographic-distance based sample pairing’) and re-filtered (PLINK --keep–geno .15 --mac 3).

#### Gene-specific subsets

##### Stomatal gene list

From the literature^31,98,99^ and lab-internal experience, we generated a list of 43 genes that are central to stomatal growth, development and the stomatal lineage (Supplementary Table 2).

##### Control gene lists

The majority of our analyses focus on per-gene statistics and are sensitive to differences in the number of SNPs, which is strongly correlated with gene length. Control genes were thus selected to match the gene length distribution of the original dataset. For each stomatal focus gene, we subsampled all genes of the same length (±2.5%), randomly picked one gene and generated a list of 43 control genes for each stomatal focus gene (up to 1,000 re-samplings per analysis).

##### Subsetting

SNPs were extracted from vcf files based on stomata-specific and control gene lists using bash and PLINK v1.90b6.16 64 bit. We filtered annotations for the first and second splice isoforms of the focus genes. SNP types (synonymous, non-synonymous, LOF and so on, based on TAIR/SnpEff annotation) were assessed based on the first splice isoform, grouping several SNP-types together: ‘loss-of-function’ (disruptive_inframe_deletion, disruptive_inframe_insertion, inframe_deletion, inframe_insertion, frameshift, start_lost, stop_lost, stop_gained), ‘non-synonymous’ (missense), ‘synonymous’ (synonymous), ‘UTR’ (5_prime_UTR_premature_start_codon_gain, 3_prime_UTR, 5_prime_UTR), ‘intron’ (intron) and ‘other’ (none, splice_region, splice_donor, splice_acceptor, intron, stop_retained, non_coding_transcript_exon, upstream_gene, downstream_gene).

### Population genetics statistics

Population genetics analyses were conducted per focus group and per gene to assess whether stomatal development genes individually or as a group are outliers. To compare stomatal developmental genes (focus genes) as a group against the control genes (‘Control gene lists’), we calculated mean values for each of the 1,000 × 43 control groups and assessed whether the stomatal gene group value was higher or lower than the majority of control group means. For per-gene assessments, we calculated mean values for each focus gene and each of 1,000 gene-specific control genes. For comparing, we calculated whether the focus gene lies outside of the 0.1 or 0.9 quantile of the control value distribution. All summary calculations, statistical analysis and plotting were done in R/RStudio.

#### Genetic diversity—${\mathbf{\uppi}}$ and SNPs per gene

We summed SNPs for each gene and calculated raw SNPs/bp (that is, per gene length) and Watterson’s *θ*. To estimate nucleotide diversity *π* per polymorphic site, we used VCFtools default settings (<--site-pi > ; VCFtools/0.1.16,^96^) and extracted the maximum per-site *π* value per gene. We also calculated $\frac{\varPi }{\rm{bp}}$ by dividing the sum of all *π* values for a single gene by the gene length (bp), assuming that positions lacking a *π* value are invariant.

#### Tajima’s *D*

To identify signals of selection in our genes of interest, we calculated Tajima’s *D*^47^ on the full 1001G vcf file (--TajimaD 100; VCFtools/0.1.16; ref. ^96^). We then associated these values to our biallelic SNPs of interest and summarized them using R and RStudio. We calculated the mean Tajima’s *D* value per gene for both length-matched control and focus gene sets.

For comparison of Tajima’s *D* between historical and modern samples, we calculated Tajima’s *D* for the independent subsets present in the geographically paired dataset (‘Joined historical and modern data’).

#### Population fixation index *F*_ST_

Population differentiation (fixation index *F*_ST)_ was estimated with Plink (plink --fst–within <groups>; PLINK v1.90b6.16 64 bit; refs. ^71,86^). Population subdivisions for the (‘modern’) 1001G dataset were based on either published, genetics-based global *A. thaliana* population structure (11 groups^49^), on accessions’ life strategies^51^ or on experienced (micro-)climates^50^. For the former, admixed individuals as defined in ref. ^36^ were excluded. For the latter two, accessions were grouped by temperature and precipitation seasonality of their geographic origins (WorldClim.org) or by their environment-independent germination rate and cold-induced dormancy (see refs. ^51,100^, excluding accessions with imputed phenotypes). Based on accessions’ silhouette scores (cluster R-library, silhouette, https://CRAN.R-project.org/package=cluster; ref. ^101^; R package version 2.1.0.) for 2 through 15 clusters, we identified and used the number of clusters with the highest score (6 and 4, respectively) in a single *k*-means clustering. Environmental and life history-based *F*_ST_ was calculated as described for stomatal genes and 1,000 control re-samplings.

Using *F*_ST_ to assess the genetic differentiation between populations not across (geographic) space but over time, we also calculated *F*_ST_ for the paired dataset (‘Joined historical and modern data’), with the samples’ identity as ‘modern’ or ‘historical’ defining the two populations.

### Matching historical and modern samples

#### Geographic-distance-based sample pairing

Historical samples were matched to the geographically closest modern sample from the 1001G *A. thaliana* dataset^36^. For each historical sample, we calculated pairwise distances to the geographic origin of all modern samples (geosphere R-library, distVincentyEllipsoid; ref. ^102^; R package version 1.5-10; https://CRAN.R-project.org/package=geosphere). We selected the closest modern sample and removed it from subsequent pairwise distance calculations. Historical–modern pairs >500 km apart were removed, generating a final dataset of 126 pairs. Historical samples from Africa and pairs between islands and mainland or matched across bodies of water or mountain ranges were excluded (Supplementary Table 6). For some analyses, samples from North America (originating west of longitude −25°) were removed, retaining 116 sample pairs. Only sample pairs with the historical collection date predating the modern one were considered.

#### Climate change trajectories

Despite this close pairing, sample pairs’ geographic origins may still be sufficiently far apart to have experienced diverging climatic changes between historical and modern sampling. This may translate into differing selection pressures and genetic makeups already in the past, which complicates attributing genetic differences between historical and modern samples to temporal (adaptive) processes alone. We reduced these confounders by modelling the directionality of climate change between 1958 and 2017 (TerraClimate dataset, resolution ~4 km; ref. ^76^). For the geographic location of each sample, we extracted precipitation and the maximum monthly temperatures (raster R package; ref. ^103^; R package version 3.4-10; https://CRAN.R-project.org/package=raster). Temperatures were then subset to one value per year, the month with the highest (*t*_max_) recorded temperature. To extract monthly precipitation trends, we transformed records into a time series and decomposed it to separate the gradual trend over time from the periodic seasonal precipitation variation (R/Rstudio, stats-package). With the annual values for *t*_max_ and precipitation per sample location, we assessed directionality (increase or decrease) of climate change by extracting the slope of a linear regression of the climate parameter over time. Using Spearman correlation, we calculated the *P* values of these climate change trends, corrected for multiple testing (Benjamini–Hochberg), and assigned trends of *P*_BH_ < 0.01 as significant. Sample pairs were then classified as matching (same significant increase or decrease in temperature or precipitation), not showing any significant change, or not matching (opposing directionalities of change, or only one sample showing a significant change in either direction).

### Stomatal density

#### Experimentally informed genetics-based stomatal density proxy

To reconstruct phenotypes from genotypes, we generated a proxy for stomatal density by summing over variants of involved genes with known functional effect. We defined the effects of mutation in the 43 stomatal genes: higher stomatal density (14 genes), lower stomatal density (10 genes) or none (18 genes; Supplementary Table 4). We then used as protein function affecting (non-synonymous, putative LOF; referred to as putatively ‘functional’) assigned SNPs (‘Gene-specific subsets’) to calculate a stomatal density proxy for each historical and modern sample. We refer to this as a ‘functional score’ to distinguish it from a traditional GWA-based polygenic risk score (‘Traditional polygenic score model’).

Accessions that carry the reference allele for a functional SNP were assigned a value of ‘0’ (no stomatal density effect). The alternative allele translates into ‘+1’ in a gene whose mutation increases, and ‘−1’ in a gene whose mutation decreases stomatal density. To calculate a density score per sample, we summed these values across all density-affecting genes, taking one functional SNP per gene into account.

The functional score correlated with published experimentally measured stomatal densities and δ^13^C (ref. ^16^). Assessment of the score’s correlation with latitude was performed on the full set of 191 historical samples. To account for geographical sampling bias, the modern 1,135 accessions were subsampled 100 times without replacement to mirror the size of the historical dataset. Linear regression was calculated for all sample sets. To avoid extrapolation beyond the geographical space covered by samples, we re-calculated the intercept using the median latitude of the historical and modern sample set, respectively. To assess the contribution of *A. thaliana*’s global population structure to the score, we permuted the association of density phenotypes (increase/decrease) with stomatal genes 100 times. This aimed to validate the stomatal density score’s latitudinal trajectory and the density shift over time. For the latter, we subtracted the historical sample’s score from its modern match within each geographic distance-based historical–modern sample pair (‘Matching historical and modern samples’). This aims to reduce population structure differences between historical and modern samples, increasing the probability that identified differences result from change over time (Supplementary Text 5).

We further subset the sample pairs by their experienced climate change (‘Climate change trajectories’). Only locations where the change from 1968 to 2017 is significant were included. With Fisher’s exact test for count data (stats::fisher.test(), R) we calculated the odds of stomatal density decrease in modern samples under certain climate conditions, identifying density score changes for each sample pair individually.

#### Traditional polygenic score model

For each of 1,000 iterations, phenotypes^16^ were randomly split 4:1 into training and test sets (scikit-learn sampling^104^). With GEMMA (v0.89.1; ref. ^105^), we calculated genome-wide associations and associations with the 43 stomata genes as described above, using a univariate linear mixed model on the training phenotypes and the joined historical and modern genotypic datasets.

We then generated an additive polygenic score (PGS, Plink (v1.9)) model on the genome-wide/stomata gene associations with the stomata phenotypes, using a *P*-score threshold <0.05 to select the most predictive SNPs. Plink and R scripts for PGS analysis followed published methods^72^. Phenotypes predicted by the PGS model were compared with the test set or with the functional stomatal density score (‘Experimentally informed genetics-based stomatal density proxy’) using one-sided Pearson’s correlation tests. To test whether the PGS models capture the phenotypes’ genetic signatures, we ran 1,000 iterations where the phenotypic data were permuted and reassigned to random genotypes in the training set and subsequently performed GWA and Plink modelling as above. For all comparisons from the 1,000 iterations, we calculated the mean predicted PGS phenotype per accession and tested these phenotypes’ correlation with measured stomatal density or the stomatal density score with one-sided Pearson’s tests.

#### Plant growth and conditions

*A. thaliana* mutant line seeds were sterilized with chlorine gas (50 ml bleach with 2.5 ml 37% hydrochloric acid) and stratified on MS plates (1/2 MS (Caisson Labs), 1% agar (*w*/*v*), pH 5.7) for 2 days at 4 °C before transfer to a 22 °C chamber with 16 h light/8 h dark cycles (110 μmol m^−2^ s^−1^).

The following mutants and transgenic lines were reported previously: *basl-2* (ref. ^106^), *epf1-1* (ref. ^53^), *epf2-1* (ref. ^107^), *ice1-D* (*scrm-D*; ref. ^54^), *ice1-2* (ref. ^54^), *mute* (ref. ^45^), *scrm2-1* (ref. ^54^), *sdd1-1* (ref. ^58^), *spch-3* (ref. ^44^), *tmm-1* (ref. ^108^), *fama* (ref. ^46^), *epf1-1;epf2-1* (ref. ^107^) and *ice1-2;scrm2-1* (ref. ^54^). Natural *A. thaliana* accessions were published previously (Col-0; for example, ref. ^36^).

#### Microscopy and image analysis

To visualize cell outlines, we stained 9 days post germination seedlings of *A. thaliana* mutant lines with FM4-64 (N-(3-triethylammoniumpropyl)-4-(6-(4-(diethylamino)phenyl)hexatrienyl)pyridinium dibromide, ThermoFisher catalogue number T13320). The *spch-3* mutant (ref. ^44^) contains a plasma membrane marker, *pATML1::mCherry-RCI2A*, and was not FM4-64 stained. Cotyledons were imaged on a Leica SP5 confocal microscope with HyD detectors using a 40× NA1.1 water objective at a resolution of 1,024 × 1,024 pixels. Images were post-processed (contrast enhancement and noise reduction) using Fiji (V2.1.0/1.53c; ref. ^109^) and Adobe Illustrator V26.3.1.

### Reporting summary

Further information on research design is available in the Nature Portfolio Reporting Summary linked to this article.

## Supplementary information

Supplementary Information Table of supplementary contents; Info on supplementary tables, Supplementary Texts 1–5 and Supplementary Figs. 1–8.

Reporting Summary

Supplementary Tables 1–12 Supplementary Tables 1–12, as described in table of contents in Supplementary Information.

## Data Availability

*A. thaliana* 1001 variant data were published in ref. ^36^ and respective kgroups in ref. ^49^. Historical sequencing data are publicly available (North American accessions^40^, African accessions^89^, German accessions^34^, broadly geographically distributed accessions^35^). Supplementary tables are available online via figshare at 10.6084/m9.figshare.25996414 (ref. ^110^).
