# Supplementary material for: Century-long timelines of herbarium genomes predict plant stomatal response to climate change
Source: Nat Ecol Evol. 2024 Aug 8;8(9):1641–53. doi: 10.1038/s41559-024-02481-x (PMC11383800; doi:10.1038/s41559-024-02481-x)
Supplement: Supplementary file 1 — Table of supplementary contents; Info on supplementary tables, Supplementary Texts 1–5 and Supplementary Figs. 1–8. [file 41559_2024_2481_MOESM1_ESM.pdf]

nature ecology & evolution

Supplementary information

https://doi.org/10.1038/s41559-024-02481-x

Century-long timelines of herbarium genomes predict plant stomatal response to climate change

In the format provided by the authors and unedited

Table of Contents

Supplementary Tables

Available for download as supplementary data .xls through the publisher.

Table S1. Historical sample information.
Table S2. Stomatal development genes.
Table S3. Presence of different SNP categories in stomatal core genes.
Table S4. Stomatal genes used for PGS.
Table S5. Correlations of whole-genome and stomatal gene PGS versus measured stomatal density and stomatal density score.
Table S6. Historical and modern sample pairs.
Table S7. Stomatal density changes across sample subsets (Student's T-test).
Table S8. Stomatal density correlation with latitude and change across sample subsets.
Table S9. Stomatal density change correlation with climate (Linear models).
Table S10. Stomatal density change correlation with climate (Fisher's Exact test).
Table S11. Stomatal density score correlation with latitude and population structure correction.
Table S12. Stomatal density score change over time with latitude and population structure correction.

Supplementary Texts

Text S1. Temporal differentiation in stomatal genes
Text S2. Stomatal density score versus stomatal index measurements
Text S3. Stomatal density score versus GWA
Text S4. Stomatal density score correlation with latitude
Text S5. Extended analyses of stomatal density trends over time and with climate

Note on physiologically-relevant temperature values

Supplementary Figures

Fig. S1: Modern and historical samples and historical sample authentication.
Fig. S2: Per-gene values of diversity statistics.
Fig. S3: Fixation index for different population delineations.
Fig. S4: Latitude patterns validate biological relevance of stomatal density score.
Fig. S5: PGS correlates with measured stomatal density and stomatal density score.
Fig. S6: Temporal change across historical and modern sample pairs.
Fig. S7: Stomatal density score decreases over time.
Fig. S8: Allele coverage support.

Supplementary Text

Text S1. Temporal differentiation in stomatal genes

We also made use of the paired historical and modern samples to calculate the sample groups' temporal differentiation, using either F_{ST}^{time} to measure differentiation between historical and modern populations, or investigating changes in Tajima's D between the two. *SCRM2*, *ERL2* and *MYB60*, outliers in one or several of the ancestry-, climate- and life-history-based F_{ST} analyses (**Fig. S3**), have F_{ST}^{time} values close to the mean or among the 10% lowest values of their respective control genes, suggesting that their differentiation following geography and climate might have been consistent over time and already present historically (see also **Fig. S6d**). In contrast, the cell-cycle gene *CYCD5;1* (AT4G37630, affects divisions in the stomatal lineage; [1]), *SCAP1* (AT5G65590, a transcription factor in the stomatal lineage; [2]) and *STOMAGEN* are highly differentiated over time, with F_{ST}^{time} among the 10% highest values of their background genes (for changes in Tajima's D between historical and modern paired samples, see **Fig. S6b,c**). It is crucial to note that the observed pattern may reflect dataset-inherent differences that are difficult to account for, such as the excess of low-frequency variants that is driving the modern data's negative mean Tajima's D (**Fig. 1d**). We aimed to address this by including only high coverage SNPs shared by both datasets addresses. However, this stringent filtering may also reduce the analyses' sensitivity, especially as the final paired dataset is small (126 sample pairs). Differences in population history and sampling-location specific climate change that remain despite the geographic sample pairing add further noise to these analyses, as does the broad time window of samples classified as "historical" (1817-2002). Nevertheless, some genes seem to show indications of change across temporal and geographic gradients. Their potential connection to climate change adaptation will however require further analyses and experimental verification.

Text S2. Stomatal density score versus stomatal index measurements

We chose to generate a polygenic score-like metric (referred to as "functional score") based on stomatal density rather than stomatal index (SI), i.e. the ratio of stomata to the total number of epidermal cells, as much fewer experimental studies have detailed the effects of loss of stomatal development genes on SI, while stomatal density is routinely measured. Notably, studying SI might to an extent alleviate some of the limitations of predicting stomatal density change accurately. While stomatal density reflects the leaves' physiological capacity and varies greatly with cell or leaf size changes, SI is independent of the latter and a direct indicator of the cellular and developmental processes generating stomata and leaf growth. As environmental changes may affect both in different ways, complementary studies can expand our understanding of which developmental pathways of plasticity and growth change affect SI, and how this modulates stomatal density and thus gas exchange. Studying SI therefore represents a promising avenue for future studies to better understand the nature of changes in stomatal development following climate change.

Text S3. Stomatal density score versus GWA

Genome-wide association (GWA) methods are typically used to map genetic variants underlying phenotypic diversity in a population without a priori candidates of the genes involved. For stomata, a previous GWA study [3] identified peaks associated with phenotypic variation.

An obvious approach to assess stomatal density changes over time based on genetic variation would be to use associations retrieved from such a stomatal density GWA, and combine them with historical genomes to project phenotypes to the past using regular GWA-based polygenic scores. However, the genes under stomatal density GWA association peaks were not connected to known stomatal genes, and need further functional validation. In addition, estimating effect sizes of SNPs can be difficult in the presence of linkage disequilibrium and population structure, which may complicate interpreting projections of phenotype change over time. We thus developed the stomatal density score as a simpler and complementary approach guided by functional information of stomatal genes. For

completeness and quality assessment, we nevertheless also conducted GWA-based polygenic scores and show that both methods produce correlated scores (see **Fig. 2f, Fig. S5c**).

Text S4. Stomatal density score correlation with latitude

A. thaliana's extensive population structure complicates disentangling demographic from adaptive genetic signatures [4]. We attempt to address this in our analyses of stomatal density score correlations with latitude by permuting genetic and phenotypic signals (see main text and **Fig. S4d,e**), and formally correcting for population structure using a genetic relationship matrix (see below).

We used the published 1001G SNP-matrix to decompose genetic relationships between the 1134 modern accessions with a principal component analysis (PCA; Plink v1.9, [5,6]). Modeling *stomatal density score ~ latitude* confirms the explanatory power of samples' latitude of origin for the stomatal density score (p-value$_{lat}$ = $<2\times10^{-16}$, $R^2_{lat} = 6.47\times10^{-2}$, **Table S11**). We also calculated latitude correlations for 100 random independent subsets of 126 modern samples. These subsets retain the correlation pattern (all slopes positive, and significant in 69 of 100 samplings; **Fig. S4a**, **b**, **Table S11**), indicating that the trend is unlikely due to geographic sampling bias.

When correcting for population structure, addition of the first three PCs marginally decreases the percentage of variance explained by the model (*stomatal density score ~ latitude + PC1 + PC2 + PC3*; p-value$_{lat}$ = $<2\times10^{-16}$, $R^2_{lat} = 6.47\times10^{-2}$, p-value$_{lat+PC123}$ = 3.00×10^{-16}, $R^2_{lat+PC123} = 6.43\times10^{-2}$, **Table S11**). Further, in this model, latitude remains the single significant coefficient (p-value$_{lat}$ = 1.42×10^{-15}, p-value$_{PC1}$ = 0.119, p-value$_{PC2}$ = 0.792, p-value$_{PC3}$ = 0.914). This is consistent also in the subset of 126 modern samples that are paired with a historical counterpart (p-value$_{lat}$ = 0.012, p-value$_{PC1}$ = 0.515, p-value$_{PC2}$ = 0.671, p-value$_{PC3}$ = 0.244), as well as in the majority of 100 random subsets of 124 modern samples (11/100 subsets with only $p_{lat} < p_{PC1}$, 25/100 $p_{lat} < p_{PC1 \mid PC2}$, 42/100 $p_{lat} < p_{PC1 \mid PC2 \mid PC3}$, i.e. a total of 78/100 with at least $p_{lat} < p_{PC1}$; of these, 51/100 have only latitude as a significant coefficient with $p < 0.05$, PC1 is significant in 18/100, of which 14 also have latitude significant, with a lower p-value for latitude than any PC in all cases). The correlation between the stomatal density score and latitude thus seems to go beyond reflecting population structure alone.

Text S5. Extended analyses of stomatal density trends over time and with climate

To rule out that the species' demographic history and thus the geographic composition of the sample pairs may be the main contributing factor to the shifts in density score (delta$_{score}$) over time (**Fig. 3, S7**; see also **Text S4**), we (1) conducted a permutation approach, (2) compared matched background controls, (3) tested the robustness of the trend by dropping samples, and (4) modeled delta$_{score}$ while accounting for genetic population structure:

First, we permute the direction of gene effects (positive vs negative) on stomatal density (see Materials and Methods: **Stomatal density**), while preserving the SNP-matrix and thus population structure. The original delta$_{score}$=-0.730 is significantly different from the mean of 100 independent such permutations (delta$_{score_permuted}$ = 0.279; Wilcoxon signed rank test, $p < 2.2\times10^{-16}$; **Fig. 3b**), and is in the extreme tail of the permuted distribution (92/100 > -0.730). For completeness, we also assessed the effect of full randomization of the SNP-matrix, retrieving as expected no consistent directionality of delta$_{score}$ trajectories (100/100 > -0.730; delta$_{score_permuted_full}$ = 0.514; Wilcoxon signed rank test, $p < 2.2\times10^{-16}$).

Second, we used background gene sets (i.e. not annotated as involved in stomata development) to generate mock temporal predictions of stomatal density score change. Assigning control genes the effect directionality of their length-matched stomata genes (approximately same number of SNPs) to calculate a mock stomatal density score again resulted in no consistent directionality of delta$_{score_mock}$ trajectories over time, with a distribution mean that is significantly different from the true delta$_{score}$ (mean delta$_{score_mock}$ = 0.760; Wilcoxon signed rank test, $p < 2.2\times10^{-16}$), where the true score lies again in the extreme tail of the control distribution (92/100 > -0.730).

Third, to directly assess the effect of geographic composition of sample pools on our analyses, we generated different subsets of the original 126 paired samples, excluding either all samples from the USA (retaining samples from above longitude -50°N; remaining n_{pairs} = 104), from the USA and Spain (latitude

> 44° N, n_{pairs} = 78), all samples from Scandinavia (latitude < 54°N, n_{pairs} = 98), or all sample pairs where the collection date of one (modern) sample is unclear (n_{pairs} = 92). In all sets, stomatal density decreased in modern samples, but the trend becomes non-significant when excluding samples from latitudes lower than 44°N (USA, Spain; $p\text{-value}_{noUS}$ = 0.368, $p\text{-value}_{noUS\_noSpain}$ = 0.113; **Table S7**, **S8**). While this could suggest that the source of the temporal signal is in the US and Spain, we refrain from jumping to this conclusion given that these large scale sample exclusions could simply cause a drop in statistical power, as non-US/non-Spain samples also display a trend of decreased stomatal score, which is however not significant for these samples alone (**Table S7**, **S8**).

Fourth, to control for genetic population structure, we fit the first three genomic principal components (PC) in a linear regression model ($delta_{score} \sim mean + latitude + PC_1 + PC_2 + PC_3$), where each predictor variable is mean-centered. In this model, the mean intercept was negative and significant (mean $delta_{score}$ = -0.73, p-value = 2.63×10^{-4}), indicating the average decrease in stomatal density score was not explained by confounders after accounting for them (**Table S12**).

We also tested whether the association we find between decreasing stomatal density and increased precipitation may be biased by population structure. Again, we find that the association is significant with $p < 0.05$ in all subsets except those excluding samples from the USA ($delta_{score} \sim precipitation_{directionality}$; see **Table S9**, and testing with Fisher's Exact test in **Table S10** and **Fig. S7e,f**), and increased precipitation is the coefficient with the strongest effect on $delta_{score}$ ($estimate_{precipitation}$ = -1.074).

In addition, we test for the effect of population structure within all subsets by using PCs as described in **Text S4**. Inclusion of any of the first three PCs makes the model non-significant across all subsets ($delta_{score} \sim precipitation_{directionality}$ p-value = 0.045; $delta_{score} \sim precipitation_{directionality} + PC_x$, p-value > 0.05, see also **Table S9**), and even in subsets where the model and effect of increased precipitation are not significant with p-value < 0.05, increased precipitation consistently has a lower p-value than any of the PCs.

In summary, across all of our tests including permutations, controls, and geographic and genetic population structure, we see a consistent decrease in stomatal density score, which generally remains significant, with some stringent corrections leading to marginal significant values across our validations.

Note on physiologically-relevant temperature values: Even combinations of precipitation and temperature change to some extent conformed to previous studies, where stomatal density did not change significantly from historical to modern samples when experiencing increased maximum temperature and decreased water availability together ($odds_{tmax\_high\_ppt\_low}$ = 0.588, $p_{tmax\_high\_ppt\_low}$ = 0.279; **Fig. S7e,f**, [7]). For increased temperature alone however, unlike published experiments (e.g. [7–9]), we only detected a weak tendency for stomatal density decrease. Such weak signals for temperature-related responses (see **Table S10**) may also result from the relatively large range of moderate temperature increases measured across our sample sites (0.7-3°C, mean 2.2°C; modeled per site using monthly maximum °C and calculating the °C increase over the recorded time frame (1958-2017) with the product of 59 years times the slope of each sites' linear regression model, only considering sites with slopes of p-values < 0.01 after Benjamini Hochberg correction). In contrast, experimentally observed density decreases, which guided our intuition of stomatal change, were conducted in laboratory growth conditions by increasing temperatures by 6-10°C (22 to 28°C in [8,9], 20 to 30°C in [7]). In agreement with our observations, others have reported diverse effects of temperature (and [CO_2]) on stomatal density (stomatal density increases, decreases and no effects), while water deficit elicited a consistent response even across species (see meta-analysis [10]).

In addition, overall climate change and plant response trends over time in the present datasets may be weakened or obfuscated by the relatively short timeframe of available high-resolution historical global climate data (1958-2017, [11]), and the generalization of all paired samples from herbaria, collected between 1817 and 1957, as "historical" and all of the 1001G accessions, collected between 1992 and 2012, as "modern". Nevertheless, we identify trends that correspond well both with experimental and historical observations of stomatal density responses to the climate variation connected with global change.

Supplemental Figures

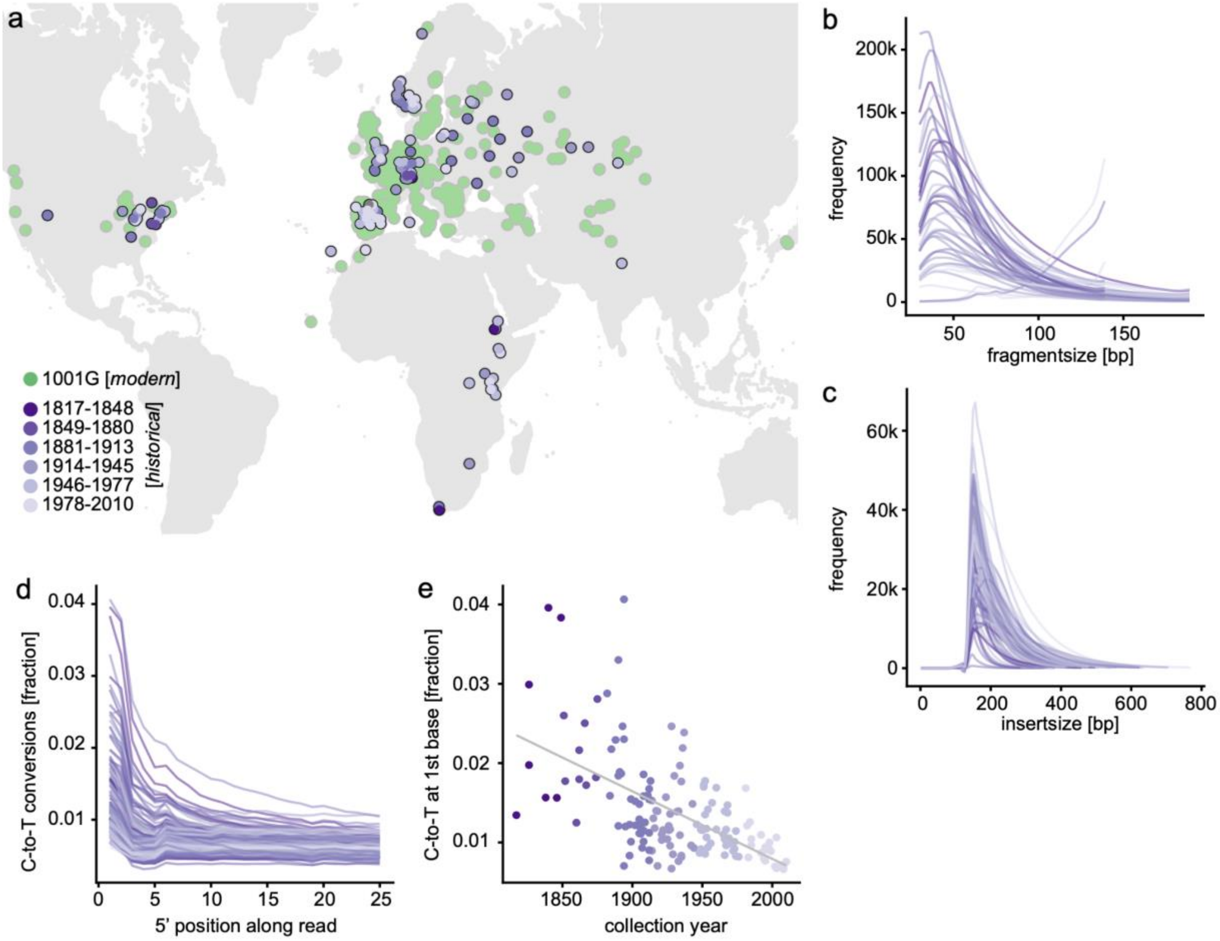

Fig. S1: Modern and historical samples and historical sample authentication. (**a**) Map of geographic origins of sequenced historical specimens, colored by sample age [12,13]. Darker purple shades indicate older samples, green visualizes geographic origin of the published 1001 *A. thaliana* genomes [4]. (**b**, **c**) Sequenced DNA fragments of historical samples, (**b**) merged sequencing reads for unsheared historical DNA and (**c**) insert sizes of sheared and unmerged DNA fragments. (**d**) Fraction of C-to-T converted base-pairs along sequencing reads, and the (**e**) correlation of the fraction of C-to-T conversion at the first base of a read with the age of the respective historical herbarium specimen (one-sided Pearson's correlation test, correlation coefficient $r = -0.589$, $p = 1.693 \times 10^{-15}$, $n = 191$).

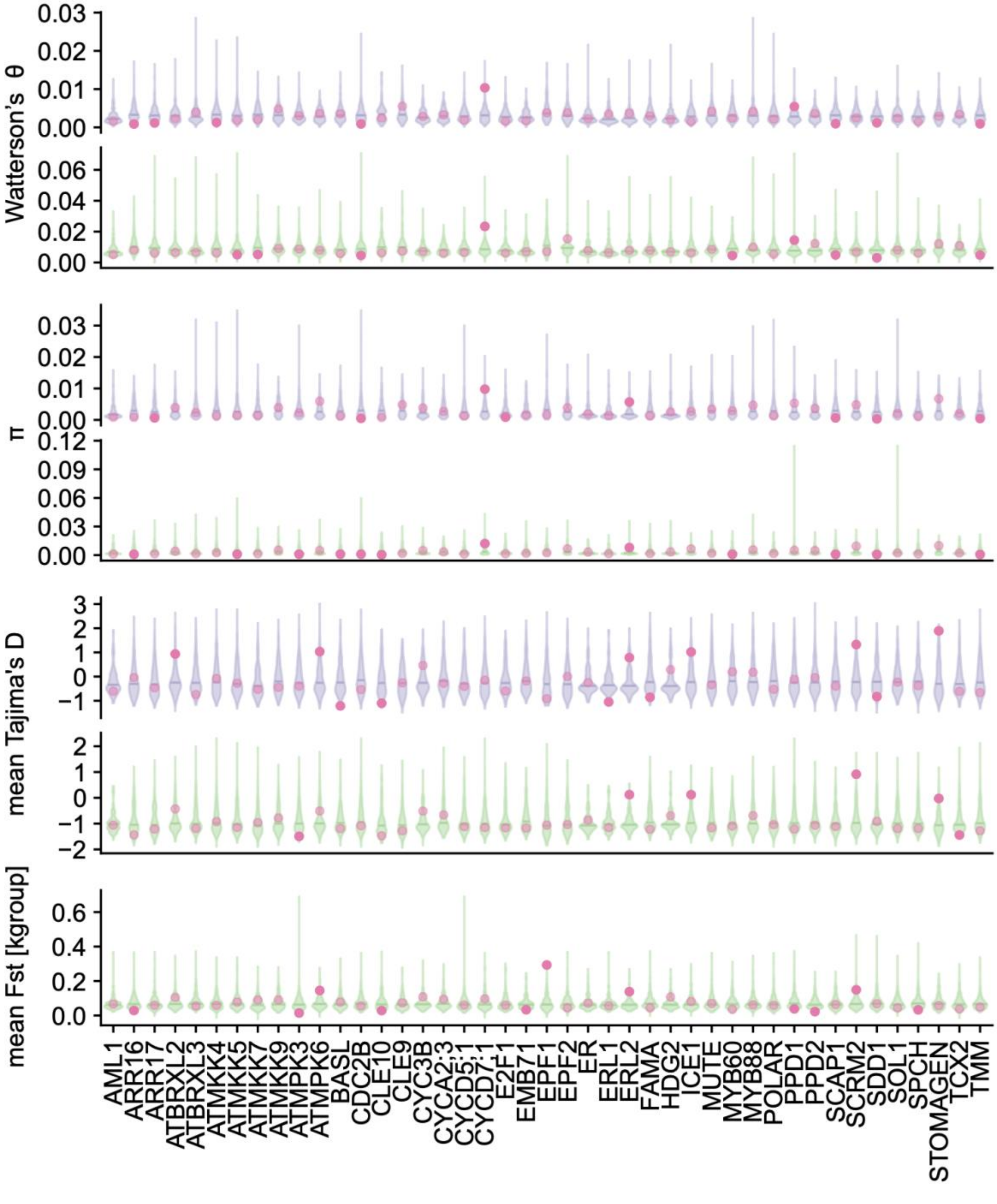

Fig. S2: Per-gene values of diversity statistics. From top to bottom, per-gene mean values for Watterson's θ, nucleotide diversity π and Tajima's D (assigned to SNPs from 100-bp-window calculations) for historical and modern samples, and for modern samples only F_{ST} (for the k-groups from [14]). Distribution of control genes of comparable length in violin plots with horizontal line marking the 0.5 percentile, purple for historical, green for modern dataset. Transparent magenta points indicate the gene mean, solid magenta points indicate that the gene mean is among the 10% lowest or highest values of the control distribution.

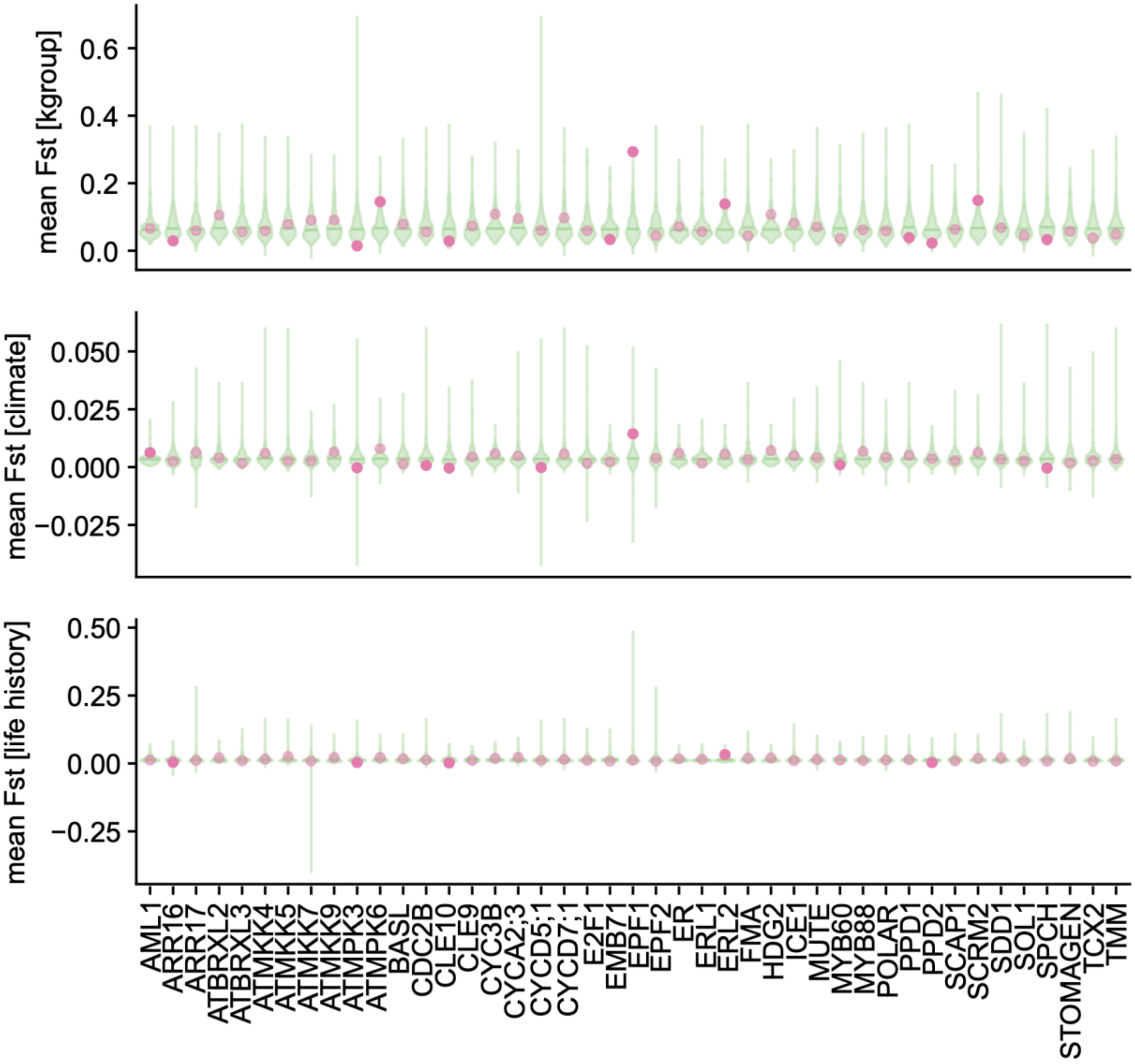

Fig. S3: Fixation index for different population delineations. Mean per-gene F_{ST} across the modern samples (green), calculated for groups defined by (from top to bottom) whole genome genetic variation ('kgroups', as defined in [14]; plot identical to **Fig. S2**, reproduced here to facilitate comparisons), climate variation (precipitation and temperature seasonality as defined in BIO4 and BIO15, [15]) and life history traits (as defined in [16]). Distribution of control genes of comparable length in violin plots with horizontal line marking the 0.5 percentile. Transparent magenta points indicate the gene mean, solid magenta points indicate that the gene mean is among the 10% lowest or highest values of the control distribution.

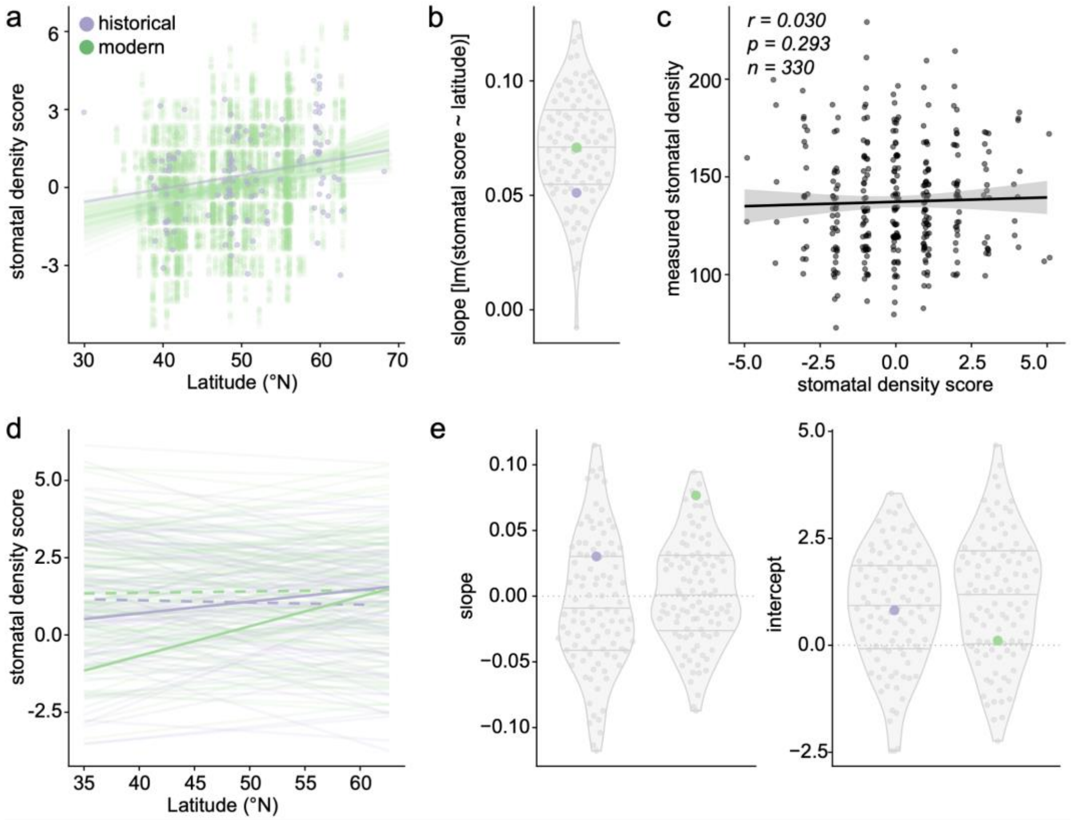

Fig. S4: Latitude patterns of stomatal density score. (**a**) Stomatal density score correlations with samples' latitudes. Historical samples (124, purple) and 100 resamplings of 124 modern samples (green), with linear regressions indicating increasing stomatal density and latitude[3]. (**b**) Slopes from regressions in (**a**), 69/100 $p < 0.05$, 100/100 positive, horizontal lines indicating 0.25, 0.5 and 0.75 quantiles. (**c**) Density score compared with measured densities (positive, not significant trend (+/- SD), linear regression slope = 0.447, one-sided Pearson's correlation test, p-value = 0.293, correlation coefficient r = 0.030, data from [3]). (**d**) Linear regressions as in (**a**), over 100 permutations of genes and their stomatal density effects (increase/decrease), historical (purple) and modern data (green). Solid lines - original data (see **Fig. 2d**, **Table S11**), transparent -permutations, dashed -permutation means. Permutation means not significantly correlated with latitude (one-sided Pearson's correlation test, $p_{mod\_mean} = 0.445$, $p_{hist\_mean} = 0.134$, correlation coefficient $r_{mod\_mean} = 0.067$, $r_{hist\_mean} = -0.134$). Linear regressions of the permuted density score with latitude are mostly not significant, and randomly positive or negative (9/100 modern and 20/100 historical significant slopes with $p < 0.05$; positive slopes in 45/100 historical and 51/100 modern regressions). (**e**) Slopes and intercepts of permutations (gray) in (**d**), original data purple (historical) and green (modern). Intercepts of linear regressions using samples' median latitude. Density differences over time in the density score-latitude association indicate a density decrease in modern samples (density score ~ latitude; y-intercept$_{modern}$ = 0.121, y-intercept$_{historical}$ = 0.799), which is supported by a linear regression integrating latitude and dataset (historical vs modern; density score ~ latitude + dataset; p-value$_{lat}$ = 0, p-value$_{dataset}$ = 0, p-value$_{model}$ = 7.732×10^{-6}). Horizontal lines indicate 0.25, 0.5 and 0.75 quantiles. Dotted horizontal lines mark slope / intercept of 0, corresponding to the expectation given full latitude independence of permuted phenotypes. The permuted samples' positive average intercept indicates a slight positive bias of the stomatal score, possibly due to the higher number of genes positively (14) vs negatively (10) affecting stomatal density used for the score's calculation. Analyses exclude North American and African samples.

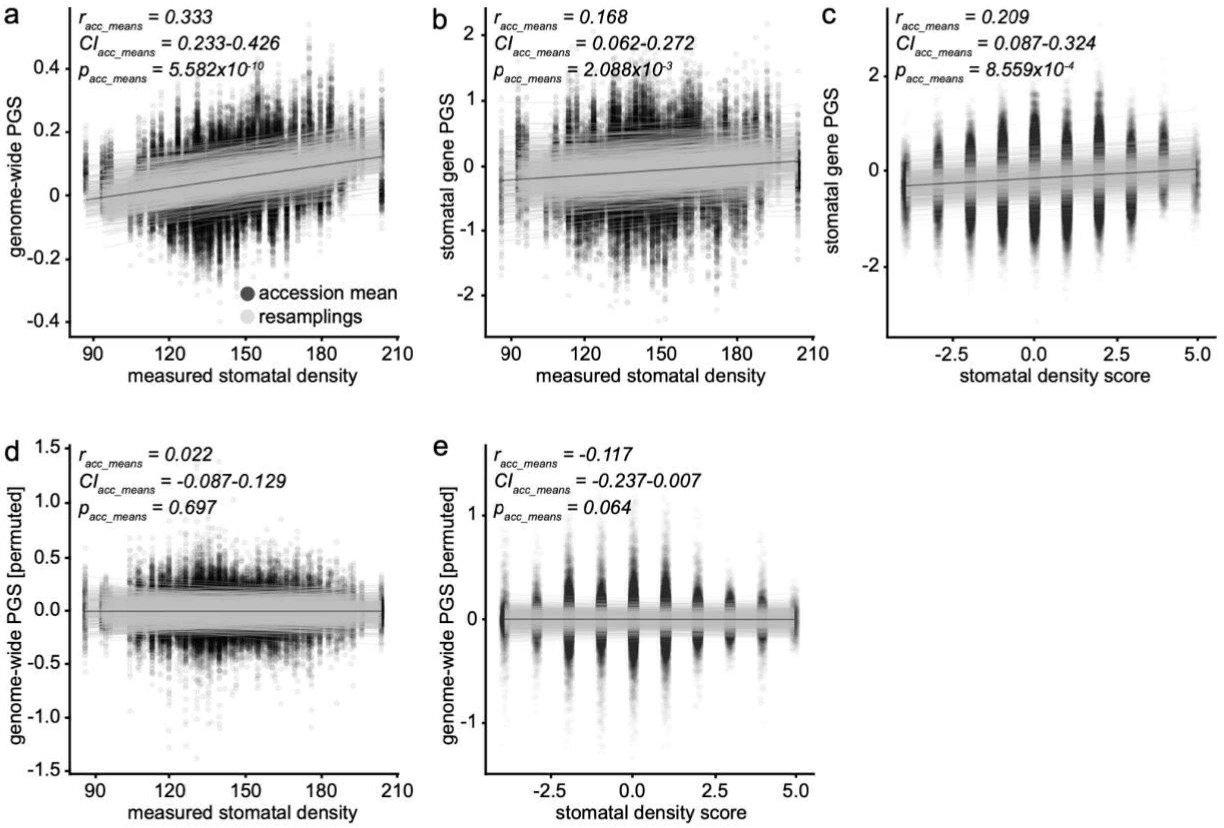

Fig. S5: PGS correlates with measured stomatal density and stomatal density score. (**a**) Genome-wide PGS correlates with experimentally measured stomatal densities [3]. One-sided Pearson's correlation test, positive correlation in 1000/1000 re-trainings, 745/1000 significant one-sided Pearson's correlation tests with p<0.05, correlation coefficient $r_{min\text{-}max}$ = 0.010-0.561; r_{median} = 0.304. (**b**) PGS generated only based on 43 stomatal development genes correlates with experimentally measured stomatal densities. One-sided Pearson's correlation test, positive correlation in 888/1000 re-trainings, 150/1000 significant one-sided Pearson's correlation tests with p<0.05, correlation coefficient $r_{min\text{-}max}$ = -0.209-0.418 , r_{median} = 0.133. (**c**) Correlation trend between stomatal gene-based PGS and the stomatal density score. One-sided Pearson's correlation test, positive correlation in 989/1000 re-trainings, 737/1000 significant one-sided Pearson's correlation tests with p<0.05, correlation coefficient r_{median} = 0.16. (**d**) Genome-wide PGS based on a training-set with permuted genotype-phenotype associations. One-sided Pearson's correlation test, positive correlation in 485/1000 re-trainings, 112/1000 significant one-sided Pearson's correlation tests with p<0.05, correlation coefficient r_{median} = -0.008. (**e**) Stomatal gene-based permuted PGS. One-sided Pearson's correlation test, positive correlation in 479/1000 re-trainings, 367/1000 significant one-sided Pearson's correlation tests with p<0.05, correlation coefficient r_{median} = -0.007. See also **Table S5**.

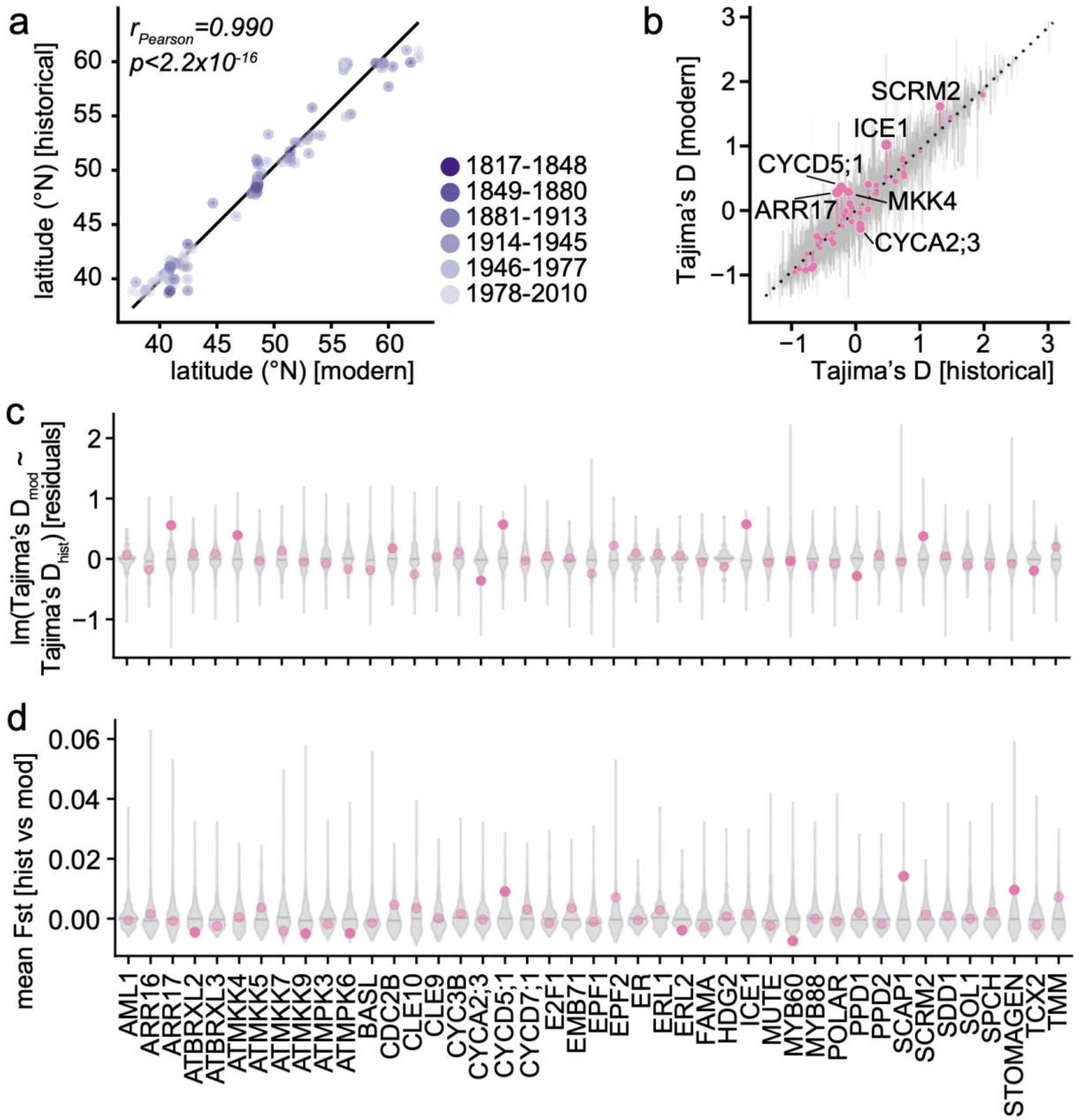

Fig. S6: Temporal change across historical and modern sample pairs. (**a**) Geographical origin-based sample pairing reduces biases in sample distribution between historical and modern datasets, as indicated by the regression of paired samples' latitudes of origin (one-sided Pearson's correlation test, correlation coefficient r = 0.990, $p < 2.2\times10^{-16}$, n = 126). (**b**) Comparison of Tajima's D values between paired historical and modern datasets, displayed as residuals of a linear regression Tajima's D_{mod} ~ Tajima's D_{hist} (dotted line). Background control genes' Tajima's D in gray, and stomatal genes' Tajima's D in magenta. Circle-size for each stomatal gene indicating the magnitude of the gene residuals' deviation from the regression. (**c**) Residuals of the linear regression model as in (**b**), per stomata gene and its specific control set. Distribution of control genes of comparable length in violin plots with horizontal line marking the 0.5 quantile. Transparent magenta points indicate the gene mean, solid magenta points indicate that the gene mean is among the 10% lowest or highest values of the control distribution. (**d**) Change in stomatal genes over time, as measured by F_{ST}^{time} between paired historical and modern sample groups.

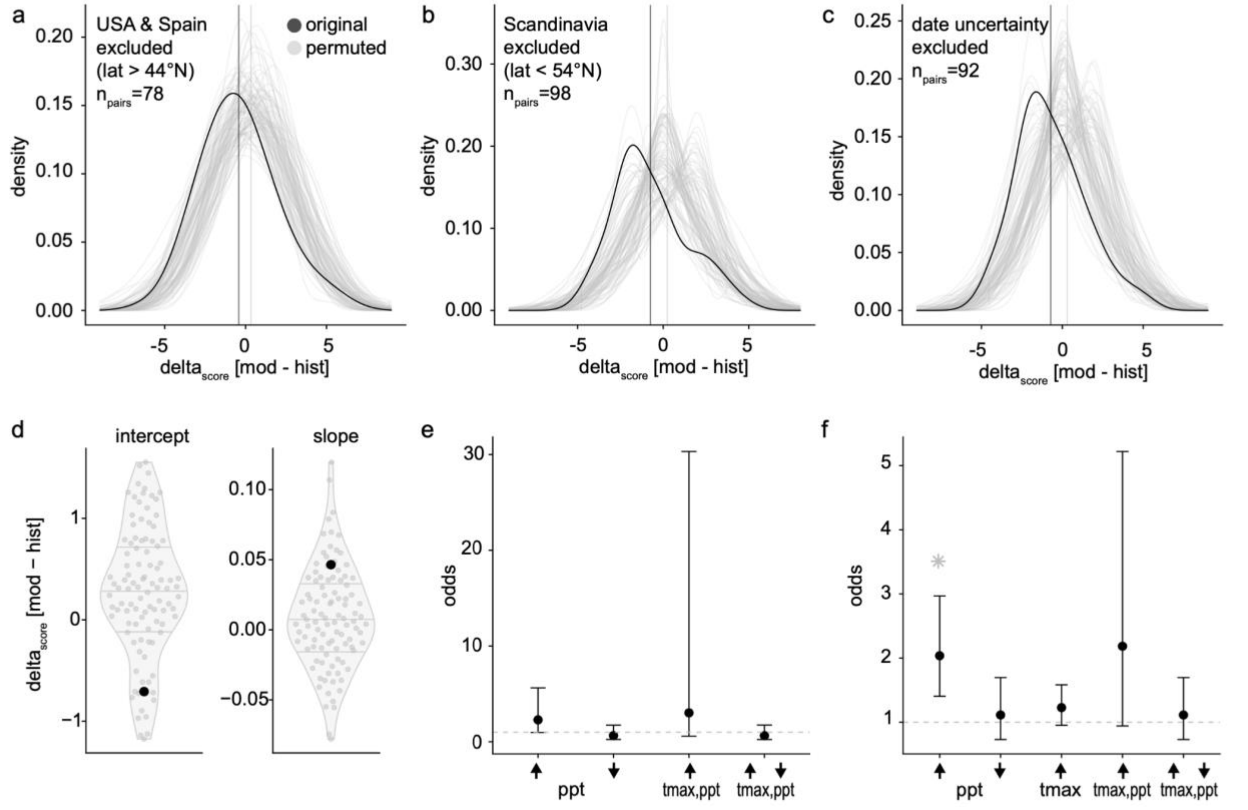

Fig. S7: Stomatal density score decreases over time. (**a-c**) Per sample-pair differences in stomatal density scores (delta$_{score}$) for original data (black) and 100 permutations (gray) of genes and their stomatal density effect (decrease/increase). Vertical lines mark density distribution means (**Table S7**, **S8)**. (**a**) Samples from latitudes >44°N, i.e. excluding the USA and Spain; mean delta$_{score}^{noUS_noSpain}$ = -0.745, smaller than 90 of 100 permutations, Wilcoxon signed rank test, $p < 2.2\times10^{-16}$. (**b**) Samples from latitudes <54°N, i.e. excluding Scandinavia; mean delta$_{score}^{noScandinavia}$ = -0.423, smaller than 93 of 100 permutations, Wilcoxon signed rank test, $p < 2.2\times10^{-16}$. (**c**) Excluding samples with uncertain collection year; mean delta$_{score}^{noUncertainty}$ = -0.717, smaller than 93 of 100 permutations, Wilcoxon signed rank test, $p < 2.2\times10^{-16}$. (**d**) Individual regressions of the density score with latitude for all historical and modern samples (black) and 100 phenotype permutations (gray). Reflects temporal change as delta$_{score}$ while accounting for remaining geographic biases. Slope and intercept were subtracted for delta$_{score}$[mod-hist]$_{slope}$ and delta$_{score}$[mod-hist]$_{intercept}$, the latter based on the median latitude of each sample group. The intercept, hinting at stomatal score decrease over time, in the original data is lower than in 89/100 permutations. The change in slope is higher than in 86/100 permutations. (**e**) Fisher's Exact Test odds of stomatal density decrease over time, calculated on historical and modern sample pairs whose geographic origin experienced a significant increase or decrease in precipitation (left), a significant increase in maximum temperature and precipitation (center), or a significant increase in maximum temperature combined with a decrease in precipitation (right) between 1958 and 2017. (**f**) Fisher's Exact Test odds of modern samples containing more SNPs that decrease stomatal density than historical samples under specific environmental conditions, independent of sample pairings. From left to right, for geographic locations with increased precipitation, decreased precipitation, higher temperature, or higher temperature and precipitation from 1958 to 2017 (Fisher's Exact test, two-sided; asterisk indicates p = 0; see **Table S10**). Error bars in (e) and (f) indicate +/-95% confidence intervals. Analyses include North American and exclude African samples.

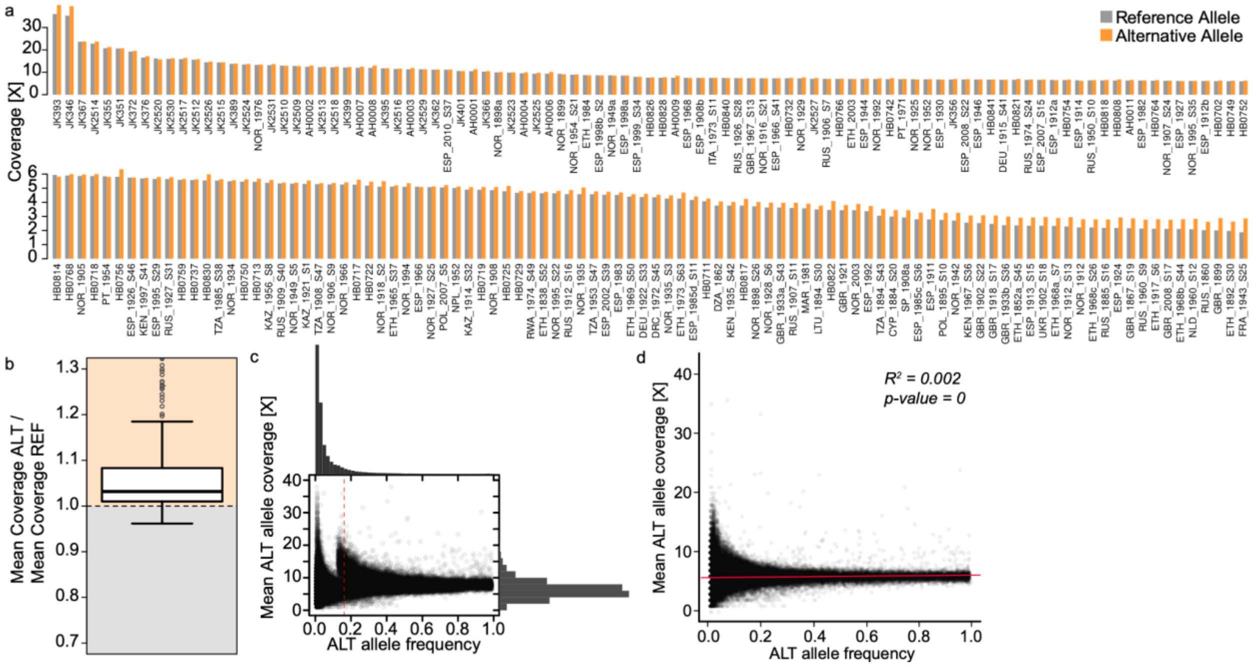

Fig. S8: Allele coverage support. (**a**) The bar plots represent the mean coverage supporting either alternative alleles (orange) or reference alleles (green) for every sample. (**b**) Summary box plot of the distribution of individual samples' values in (**a**) as a ratio of the mean coverages for alternative alleles by the mean coverages for reference alleles. Ratio values over 1 (orange area) represent samples for which alternative allele calls are supported by a higher number of reads relative to reference allele calls. (**c**) Relationship between alternative allele frequency (X axis) and the mean coverage supporting alternative alleles (Y axis) in the full Arabidopsis thaliana dataset. Histograms represent the distribution of the data for each axis. (**d**) Relationship between alternative allele frequency (X axis) and the mean coverage supporting alternative alleles (Y axis) in the dataset excluding 32 HPG1 lineage samples (linear model, p-value = 0, coefficient of determination R2 = 0.002).

References

1. Han, S.-K. *et al.* MUTE Directly Orchestrates Cell-State Switch and the Single Symmetric Division to Create Stomata. *Dev. Cell* **45**, 303–315.e5 (2018).
2. Castorina, G., Fox, S., Tonelli, C., Galbiati, M. & Conti, L. A novel role for STOMATAL CARPENTER 1 in stomata patterning. *BMC Plant Biol.* **16**, 172 (2016).
3. Dittberner, H. *et al.* Natural variation in stomata size contributes to the local adaptation of water-use efficiency in Arabidopsis thaliana. *Mol. Ecol.* (2018) doi:10.1111/mec.14838.
4. 1001 Genomes Consortium. 1,135 Genomes Reveal the Global Pattern of Polymorphism in Arabidopsis thaliana. *Cell* **166**, 481–491 (2016).
5. Purcell, S. *et al.* PLINK: a tool set for whole-genome association and population-based linkage analyses. *Am. J. Hum. Genet.* **81**, 559–575 (2007).
6. Chang, C. C. *et al.* Second-generation PLINK: rising to the challenge of larger and richer datasets. *Gigascience* **4**, 7 (2015).
7. Vile, D. *et al.* Arabidopsis growth under prolonged high temperature and water deficit: independent or interactive effects? *Plant Cell Environ.* **35**, 702–718 (2012).
8. Lau, O. S. *et al.* Direct Control of SPEECHLESS by PIF4 in the High-Temperature Response of Stomatal Development. *Curr. Biol.* **28**, 1273–1280.e3 (2018).
9. Crawford, A. J., McLachlan, D. H., Hetherington, A. M. & Franklin, K. A. High temperature exposure increases plant cooling capacity. *Curr. Biol.* **22**, R396–7 (2012).
10. Yan, W., Zhong, Y. & Shangguan, Z. Contrasting responses of leaf stomatal characteristics to climate change: a considerable challenge to predict carbon and water cycles. *Glob. Chang. Biol.* **23**, 3781–3793 (2017).
11. Abatzoglou, J. T., Dobrowski, S. Z., Parks, S. A. & Hegewisch, K. C. TerraClimate, a high-resolution global dataset of monthly climate and climatic water balance from 1958-2015. *Sci Data* **5**, 170191 (2018).
12. Lopez, L., Marciniak, S., Perry, G. H. & Lasky, J. R. *Historical Arabidopsis Thaliana Genomes from across Its Native Range*. (2022). doi:10.5281/zenodo.7187528.
13. Latorre, S. M., Lang, P. L. M. & Burbano, H. A. *Historical Arabidopsis Thaliana Genomes from Germany*. (2022). doi:10.5281/zenodo.7156189.
14. Exposito-Alonso, M. *et al.* Genomic basis and evolutionary potential for extreme drought adaptation in Arabidopsis thaliana. *Nat Ecol Evol* **2**, 352–358 (2018).
15. Hijmans, R. J., Cameron, S. E. & Parra, J. L. Very high resolution interpolated climate surfaces for global land areas. *Aquat. Microb. Ecol.* (2005).
16. Exposito-Alonso, M. Seasonal timing adaptation across the geographic range of Arabidopsis thaliana. *Proceedings of the National Academy of Sciences of the United States of America* vol. 117 9665–9667 (2020).
