# Supplementary material for: Century-long timelines of herbarium genomes predict plant stomatal response to climate change
Source: Nat Ecol Evol. 2024 Aug 8;8(9):1641–53. doi: 10.1038/s41559-024-02481-x (PMC11383800; doi:10.1038/s41559-024-02481-x)
Supplement: Supplementary file 2 — Reporting Summary [file 41559_2024_2481_MOESM2_ESM.pdf]

nature portfolio

Corresponding author(s): Patricia Lang

Last updated by author(s): Jun 13, 2024

Reporting Summary

Nature Portfolio wishes to improve the reproducibility of the work that we publish. This form provides structure for consistency and transparency in reporting. For further information on Nature Portfolio policies, see our Editorial Policies and the Editorial Policy Checklist.

Statistics

For all statistical analyses, confirm that the following items are present in the figure legend, table legend, main text, or Methods section.

n/a	Confirmed	
☐	☒	The exact sample size (*n*) for each experimental group/condition, given as a discrete number and unit of measurement
☐	☒	A statement on whether measurements were taken from distinct samples or whether the same sample was measured repeatedly
☐	☒	The statistical test(s) used AND whether they are one- or two-sided<br>*Only common tests should be described solely by name; describe more complex techniques in the Methods section.*
☐	☒	A description of all covariates tested
☐	☒	A description of any assumptions or corrections, such as tests of normality and adjustment for multiple comparisons
☐	☒	A full description of the statistical parameters including central tendency (e.g. means) or other basic estimates (e.g. regression coefficient) AND variation (e.g. standard deviation) or associated estimates of uncertainty (e.g. confidence intervals)
☐	☒	For null hypothesis testing, the test statistic (e.g. *F*, *t*, *r*) with confidence intervals, effect sizes, degrees of freedom and *P* value noted<br>*Give P values as exact values whenever suitable.*
☒	☐	For Bayesian analysis, information on the choice of priors and Markov chain Monte Carlo settings
☒	☐	For hierarchical and complex designs, identification of the appropriate level for tests and full reporting of outcomes
☐	☒	Estimates of effect sizes (e.g. Cohen's *d*, Pearson's *r*), indicating how they were calculated

Our web collection on statistics for biologists contains articles on many of the points above.

Software and code

Policy information about availability of computer code

Data collection

Modern genomic data was downloaded from http://1001genomes.org/data/GMI-MPI/releases/v3.1/.

The Arabidopsis thaliana reference genome was downloaded from https://www.arabidopsis.org/download_files/Genes/TAIR10_genome_release/TAIR10_chromosome_files/TAIR10_chr_all.fas.gz, and gene annotations from https://www.arabidopsis.org/download_files/Genes/TAIR10_genome_release/TAIR10_gff3/TAIR10_GFF3_genes_transposons.gff.

Novel German historical samples are available at GenBank, BioProject number PRJNA887392 (https://www.ncbi.nlm.nih.gov/bioproject/PRJNA887392).
Novel global samples are available upon request (https://zenodo.org/record/7187528).
North American accessions were downloaded from the European Nucleotide Archive, study PRJEB24619 (https://www.ebi.ac.uk/ena/data/view/PRJEB24619).
African accessions were downloaded from the European Nucleotide Archive/Sequence Read Archive database, study PRJEB19780 (https://www.ebi.ac.uk/ena/browser/view/PRJEB19780).

Climate data was downloaded from https://climate.northwestknowledge.net/TERRACLIMATE-DATA/.

Stomata phenotypes and water use efficiency data was downloaded from https://onlinelibrary.wiley.com/action/downloadSupplement?doi=10.1111%2Fmec.14838&file=mec14838-sup-0003-TableS2.csv (https://onlinelibrary.wiley.com/doi/10.1111/mec.14838).

Arabidopsis thaliana microscopy images were collected with a Leica SP5 confocal microscope and the Leica Application Suite Advanced Fluorescence 2.7.3.9723 and processed with Fiji v.2.9.0 and Adobe Illustrator 27.0.1.

Data analysis

All statistical analysis was conducted using R v1.2.1335 within RStudio v1.2.1335, with the packages cluster v2.1.0., geosphere v1.5-10, raster v3.4-10.

Phenotype data was processed using Python 3.7.9, with the packages pandas v1.1.2, numpy v1.19.2, and scikit-learn 1.2.0.

Genome sequence data was processed and analyzed using
Adapterremoval v2.3.1, BCFtools v1.10.2, BWA v0.7.15-r1140, DeDup v0.12.8, gatk4-4.2.0.0.-0, GEMMA v0.89.1, MapDamage v2.2.1, Picard v2.18.29-0, PLINK v1.90b6.16 64-bit, Samtools v1.9, SnpEff v5.0e, vcflib/20161123-git, VCFtools/0.1.16.

Microscopy images were post-processed with Fiji v2.1.0/1.53c and Adobe Illustrator v26.3.1.

For manuscripts utilizing custom algorithms or software that are central to the research but not yet described in published literature, software must be made available to editors and reviewers. We strongly encourage code deposition in a community repository (e.g. GitHub). See the Nature Portfolio guidelines for submitting code & software for further information.

Data

Policy information about availability of data

All manuscripts must include a data availability statement. This statement should provide the following information, where applicable:

- Accession codes, unique identifiers, or web links for publicly available datasets
- A description of any restrictions on data availability
- For clinical datasets or third party data, please ensure that the statement adheres to our policy

Data availability statement included.

Research involving human participants, their data, or biological material

Policy information about studies with human participants or human data. See also policy information about sex, gender (identity/presentation), and sexual orientation and race, ethnicity and racism.

Reporting on sex and gender

Use the terms sex (biological attribute) and gender (shaped by social and cultural circumstances) carefully in order to avoid confusing both terms. Indicate if findings apply to only one sex or gender; describe whether sex and gender were considered in study design; whether sex and/or gender was determined based on self-reporting or assigned and methods used.
Provide in the source data disaggregated sex and gender data, where this information has been collected, and if consent has been obtained for sharing of individual-level data; provide overall numbers in this Reporting Summary. Please state if this information has not been collected.
Report sex- and gender-based analyses where performed, justify reasons for lack of sex- and gender-based analysis.

Reporting on race, ethnicity, or other socially relevant groupings

Please specify the socially constructed or socially relevant categorization variable(s) used in your manuscript and explain why they were used. Please note that such variables should not be used as proxies for other socially constructed/relevant variables (for example, race or ethnicity should not be used as a proxy for socioeconomic status).
Provide clear definitions of the relevant terms used, how they were provided (by the participants/respondents, the researchers, or third parties), and the method(s) used to classify people into the different categories (e.g. self-report, census or administrative data, social media data, etc.)
Please provide details about how you controlled for confounding variables in your analyses.

Population characteristics

Describe the covariate-relevant population characteristics of the human research participants (e.g. age, genotypic information, past and current diagnosis and treatment categories). If you filled out the behavioural & social sciences study design questions and have nothing to add here, write "See above."

Recruitment

Describe how participants were recruited. Outline any potential self-selection bias or other biases that may be present and how these are likely to impact results.

Ethics oversight

Identify the organization(s) that approved the study protocol.

Note that full information on the approval of the study protocol must also be provided in the manuscript.

Field-specific reporting

Please select the one below that is the best fit for your research. If you are not sure, read the appropriate sections before making your selection.

☒ Life sciences ☐ Behavioural & social sciences ☐ Ecological, evolutionary & environmental sciences

For a reference copy of the document with all sections, see nature.com/documents/nr-reporting-summary-flat.pdf

Life sciences study design

All studies must disclose on these points even when the disclosure is negative.

Sample size	Depending on the analysis in question (see 'Data exclusions'), we used a full or partial panel of the 1001 Genomes Project.<br>We combined all available whole genome sequences of historical Arabidopsis thaliana herbarium specimens and used all or parts of this combined data as described (see 'Data exclusions').
Data exclusions	For geographic analyses, only samples with known geographic coordinates were considered.<br>For collection-time related analyses, only samples with known collection year were considered.<br><br>To compare historical with modern samples, samples were matched by geographic proximity, excluding historical-modern sample pairs with a distance larger than 500km, excluding historical samples from the African continent, and excluding sample pairs between islands and mainland, or pairs matched across bodies of water or mountain ranges. Only sample pairs with the historical collection date predating the modern collection date were considered. For comparative analyses, only subsets of samples were used as indicated in the Materials and Methods.<br><br>For the historical SNPcall, a number of quality filters were employed. Only samples with missing call frequencies smaller 50% were retained. Subsequently, we only considered called nucleotide variants with a minor allele present in at least three individual samples and with a site missingness <15%. Comparisons between historical and modern samples were conducted only on fully overlapping datasets.
Replication	Replication was not relevant in this study, as no new data was created experimentally.<br><br>All analyses were conducted with analysis-specific, replicated control datasets. For each analyzed gene, 1000 control genes were selected (randomly, with replacement) to match gene lengths of original genes. For gene-group analyses, we calculated mean values for each of the 1000 random replicates to then compare to the gene-group mean of interest.
Randomization	Randomization was not relevant in this study, as no new data was created experimentally.
Blinding	Blinding was not relevant in this study, as now new data was created experimentally.

Reporting for specific materials, systems and methods

We require information from authors about some types of materials, experimental systems and methods used in many studies. Here, indicate whether each material, system or method listed is relevant to your study. If you are not sure if a list item applies to your research, read the appropriate section before selecting a response.

Materials & experimental systems

n/a	Involved in the study	
☒	☐	Antibodies
☒	☐	Eukaryotic cell lines
☒	☐	Palaeontology and archaeology
☒	☐	Animals and other organisms
☒	☐	Clinical data
☒	☐	Dual use research of concern
☐	☒	Plants

Methods

n/a	Involved in the study	
☒	☐	ChIP-seq
☒	☐	Flow cytometry
☒	☐	MRI-based neuroimaging

Dual use research of concern

Policy information about dual use research of concern

Hazards

Could the accidental, deliberate or reckless misuse of agents or technologies generated in the work, or the application of information presented in the manuscript, pose a threat to:

No	Yes	
☒	☐	Public health
☒	☐	National security
☒	☐	Crops and/or livestock
☒	☐	Ecosystems
☒	☐	Any other significant area

Experiments of concern

Does the work involve any of these experiments of concern:

No	Yes	
☒	☐	Demonstrate how to render a vaccine ineffective
☒	☐	Confer resistance to therapeutically useful antibiotics or antiviral agents
☒	☐	Enhance the virulence of a pathogen or render a nonpathogen virulent
☒	☐	Increase transmissibility of a pathogen
☒	☐	Alter the host range of a pathogen
☒	☐	Enable evasion of diagnostic/detection modalities
☒	☐	Enable the weaponization of a biological agent or toxin
☒	☐	Any other potentially harmful combination of experiments and agents

Plants

Seed stocks

The following mutants and transgenic lines utliized for microscopy in this study were all reported previously: basl-2 (Dong et al. 2009), epf1-1 (Hara et al. 2007), epf2-1 (Hunt & Gray 2009), ice1-D (scrm-D, Kanaoka et al. 2008), ice1-2 (Kanaoka et al. 2008), mute (Pillitteri et al. 2007), scrm2-1 (Kanaoka et al. 2008), sdd1-1 (Berger & Altmann 2000), spch-3 (MacAlister et al. 2007), tmm-1 (Yang & Sack 1995), fama (Ohashi-Ito & Bergmann 2006), epf1-1;epf2-1 (Hunt & Gray 2009), and ice1-2;scrm2-1 (Kanaoka et al. 2008). Natural A. thaliana accessions were published previously (Col-0, e.g. (1001 Genomes Consortium 2016)).

Novel plant genotypes

No novel plant genotypes were produced in this study.

Authentication

No novel plant genotypes were produce in this study, and all stable seed stock lines used for microscopy imaging were published previously. No authentication was required for any lines utilized.
